# Thermal Time, Biomass Allocation and Morphometric Associations with Floral Biomass in Medicinal *Cannabis sativa* L. S_1_ Progenies Under Tropical Conditions

**DOI:** 10.3390/plants15213400

**Published:** 2026-11-02

**Authors:** Renato de T. Tonini, Matheus M. Fernandes, Lucas U. L. Soares, Thales de O. V. Ker, Aloísio F. S. Ribeiro, Heitor M. Teixeira, Derly J. H. da Silva

**Affiliations:** 1 Department of Agronomy, Federal University of Viçosa, Viçosa 36570-900, MG, Brazil; tonini@cannabreedbrasil.com (R.d.T.T.); marrazzocontato@gmail.com (M.M.F.); lucas.ulyssea@ufv.br (L.U.L.S.);; 2 Cannabreed Technology Brasil LTDA, Viçosa 36570-087, MG, Brazil; 3 Department of Soils, Federal University of Viçosa, Viçosa 36570-900, MG, Brazil; heitor.teixeira@ufv.br

**Keywords:** *Cannabis sativa* L., growing degree days, biomass partitioning, harvest index, tropical cultivation

## Abstract

Medicinal *Cannabis sativa* L. cultivation is expanding in tropical regions, although agronomic reference values largely derive from temperate or controlled environments. We assessed growing degree day (GDD)-dependent biomass accumulation, partitioning, and morphometric associations in three photoperiod-sensitive S_1_ progenies (Purple Wreck, Doctor, and Schanti). Plants grown under a 20-h photoperiod were transferred outdoors to natural daylength. Organ dry biomass was sampled weekly from 210 plants and fitted with three-parameter log-logistic models. Under a common slope, progeny-specific half-maximum parameters did not significantly improve model fit, whereas heteroscedasticity-consistent tests identified asymptote differences for stems, leaves, and roots. The Doctor-derived progeny showed the highest harvest index, despite no significant difference in final floral biomass among progenies. The pooled model estimated half-maximal floral biomass at approximately 1946 GDD. These thresholds depend on the greenhouse-to-field transition and temperature-measurement conditions; later milestones were projected beyond the sampled period. Plant height and basal stem diameter were strongly associated with total biomass, and crown diameter with floral biomass, supporting their evaluation as non-destructive predictors; when GDD was accounted for, four descriptors increased explained floral-biomass variation by at most 0.6 percentage points. These findings provide context-dependent thermal-time references for medicinal cannabis under tropical conditions, requiring validation across environments, seasons, and genotypes.

## 1. Introduction

The expansion of medicinal cannabis (*Cannabis sativa* L.) cultivation and associated markets [1] has intensified the demand for agronomic information tailored to local growing conditions. Growth and flowering dynamics in this species are influenced by sowing date and climatic conditions [2]; however, most available agronomic reference values and crop growth models have been developed under temperate climatic conditions or in controlled indoor cultivation systems [3,4,5]. Commercial hemp cultivars are predominantly of temperate origin, and at near-equatorial latitudes, where daylength remains approximately 12 h throughout the year, photoperiod-sensitive cultivars may undergo premature flowering, resulting in reduced crop yields [6]. The applicability of these findings to tropical field cultivation remains uncertain, particularly regarding growth dynamics and biomass partitioning under local photoperiods.

Thermal-time approaches based on growing degree days (GDDs) characterize plant development in terms of accumulated thermal exposure rather than calendar time, provided that crop-specific base (*Tb*) and ceiling (*Tc*) temperature thresholds and a well-defined calculation method are used [7,8]. As these parameters vary across crops, developmental stages, cultivars, and estimation methods, crop- and context-specific parameterization is required [8]. In *Humulus lupulus* L., the closest cultivated relative of *Cannabis sativa* L. within the Cannabaceae, GDD has been employed to characterize vegetative development and phenological transitions under semi-arid, subtropical, and Mediterranean conditions, with both the selected *Tb* and calculation method influencing model fit [9,10]. In other crops, thermal-time-based models have successfully described phenological progression and organ-specific biomass accumulation [11], whereas harvest index has been shown to vary with genotype [12] and temperature [13]. Thus, thermal time provides a quantitative framework for characterizing developmental progression, while biomass partitioning requires independent assessment.

Historically, phenological research on *Cannabis sativa* L. has focused predominantly on fiber hemp in temperate regions [14,15,16], emphasizing leaf appearance, canopy establishment, and phenology, while providing limited insight into floral-biomass accumulation in high-cannabinoid medicinal genotypes. Previous studies have also demonstrated that temperature affects carbon assimilation in the species: net photosynthesis peaks near 30 °C and declines at higher leaf temperatures, with responses differing between drug- and fiber-type varieties [17,18]. Under tropical daylengths, temperature and photoperiod jointly influence time to flowering, biomass accumulation, and cannabinoid concentration, with their relative importance depending on the variety [19]. For medicinal cannabis, organ biomass has been modeled on a thermal-time scale in a temperate-latitude greenhouse [5], whereas comparable field data at tropical latitudes remain limited.

In medicinal cannabis, the harvested product is the inflorescence, which bears cannabinoid-producing glandular trichomes [20]. Floral biomass and sink strength differ among genotypes and chemovars grown under the same conditions [21,22], as demonstrated by a comparison of two drug-type chemovars in which the Δ9-tetrahydrocannabinol (THC)-dominant chemovar produced twice the floral biomass of the cannabidiol (CBD)-dominant chemovar [23]. Absolute floral biomass and the proportion of biomass allocated to inflorescences, as quantified by the harvest index, therefore describe different aspects of productive performance, both of which are relevant when comparing plant material intended for medicinal use.

The test guidelines of the International Union for the Protection of New Varieties of Plants (UPOV) for *Cannabis sativa* L. [24] encompass the different end uses of the species, but descriptors specific to medicinal inflorescence traits remain limited, and morphometric relationships enabling non-destructive estimation of floral biomass have rarely been examined in this crop. In a study involving 23 hemp families, basal stem diameter was identified as the best individual selection criterion for floral dry biomass, with stem diameter, canopy kite area and plant height jointly accounting for 67% of the explainable variation in floral yield [25]. In an evaluation of 121 medicinal genotypes grown under controlled conditions, vegetative-stage measurements yielded a prediction equation for floral bud dry weight (adjusted R^2^ = 0.34) that could be used to rank candidate genotypes before the end of the cycle [26]. Morphometric traits may also vary throughout plant development. In a drug-type cultivar, leaf morphology changed along successive nodes, with leaflet number and leaf area first increasing and then decreasing over the life cycle [27]. Consequently, relationships between plant dimensions and biomass measured across sampling dates incorporate growth-related variation. Their potential for estimating biomass across the crop cycle should therefore be distinguished from their ability to discriminate among plants at a common sampling stage. 

Three photoperiod-sensitive S_1_ (first selfed generation) progenies derived from medicinal cultivars were grown under a 20-h photoperiod in a greenhouse and then transferred to the field under the natural photoperiod of a tropical latitude. We examined whether the progenies differed in fitted growth timing, expressed as the GDD at half-maximal biomass, and in the magnitude of biomass accumulation and reproductive allocation, expecting similar timing under the shared management sequence but differences in magnitude and allocation. The objectives were to (i) describe growth and the partitioning of biomass among organs as a function of GDD, (ii) characterize the progression of harvest index, and (iii) assess the associations of morphometric descriptors, including UPOV traits, with total and floral biomass as candidate equations for non-destructive biomass estimation. A complementary analysis examined whether four descriptors added information on floral biomass beyond accumulated GDD during reproductive development.

## 2. Results

### 2.1. Growth Conditions

**Figure 1:**
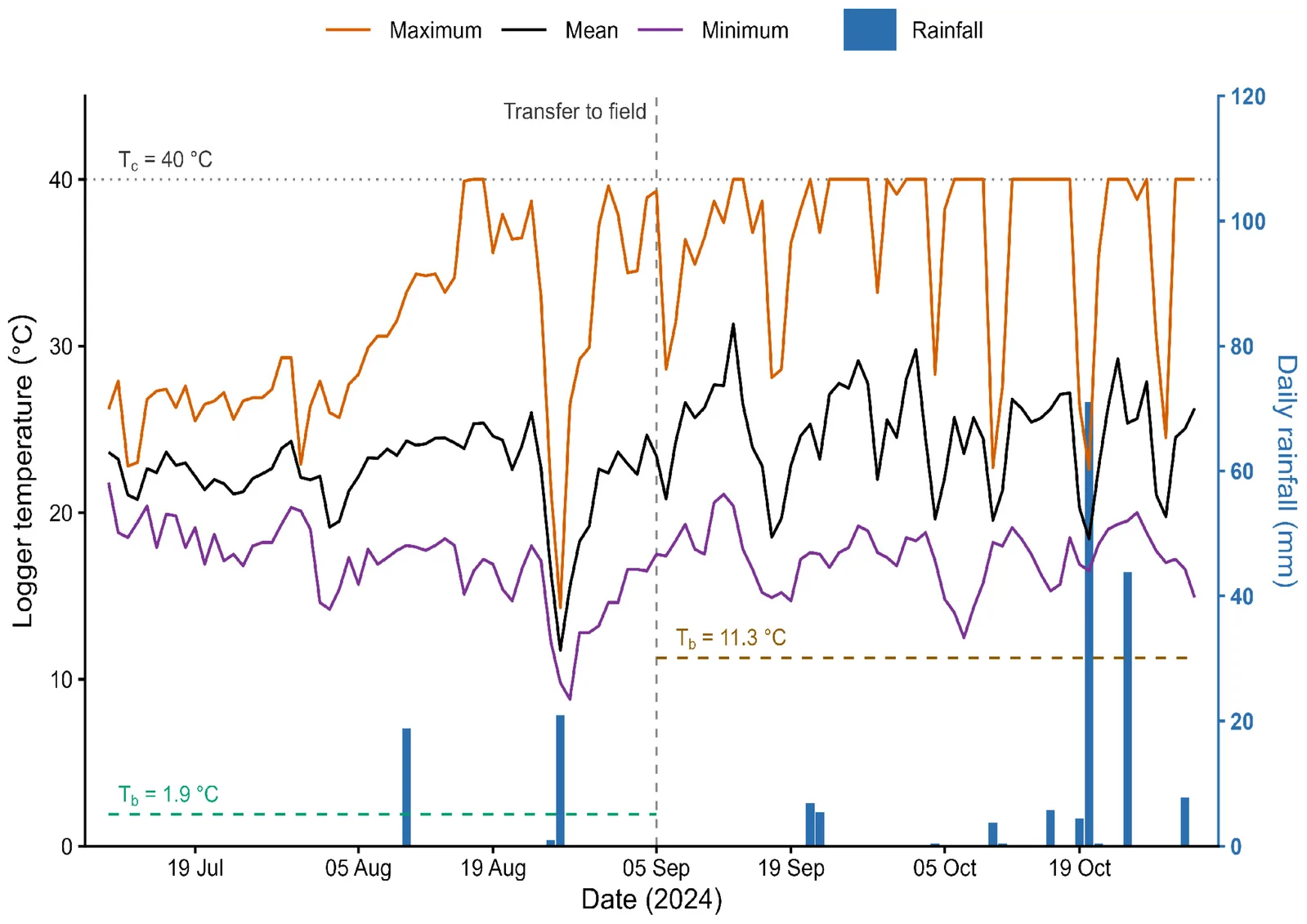
Daily maximum, mean, and minimum temperatures recorded by the sheltered data logger (left axis) and rainfall at the INMET A637 station (right axis), 10 July–31 October 2024. Maximum temperatures were capped at *Tc* = 40 °C (dotted line) for GDD calculations (Section 4.5). The vertical dashed line indicates transfer to the field (5 September 2024), and the horizontal dashed lines indicate the base temperatures (*Tb*): 1.9 °C (green) before transfer and 11.3 °C (brown) thereafter.

The experimental period extended from winter to spring (10 July–31 October 2024), with increasing daylength from August to October. Monthly mean temperatures recorded at the experimental site were 22.4 °C in July (from 10 July), 22.2 °C in August, 24.9 °C in September, and 24.6 °C in October. The lowest recorded temperature in each month was 16.8, 8.8, 14.6, and 12.5 °C, respectively. The mean daily temperature range increased from 17.1 °C in August to 21.1 °C in September and 23.6 °C in October. Figure 1 presents daily temperatures recorded at the experimental site by the data logger used for thermal-time calculations (Section 4.5), alongside daily rainfall measured at the nearest station of the Brazilian National Institute of Meteorology (INMET) [28].

Recorded daily maximum temperatures reached or exceeded the 40 °C ceiling temperature on 32 of the 114 experimental days, including 30 days after transfer to the field. For regional meteorological context, mean hourly temperatures at the INMET station were 17.2 °C in August, 21.2 °C in September, and 21.9 °C in October, with hourly extremes of 4.2 °C in August and 36.2 °C in October.

Monthly rainfall was 41.0 mm in August and 12.4 mm in September, then rose to 138.2 mm in October with the onset of the rainy season, including 71.0 mm on 20 October and 43.8 mm on 24 October. 

### 2.2. Thermal Development and Phenological Thresholds

**Figure 2:**
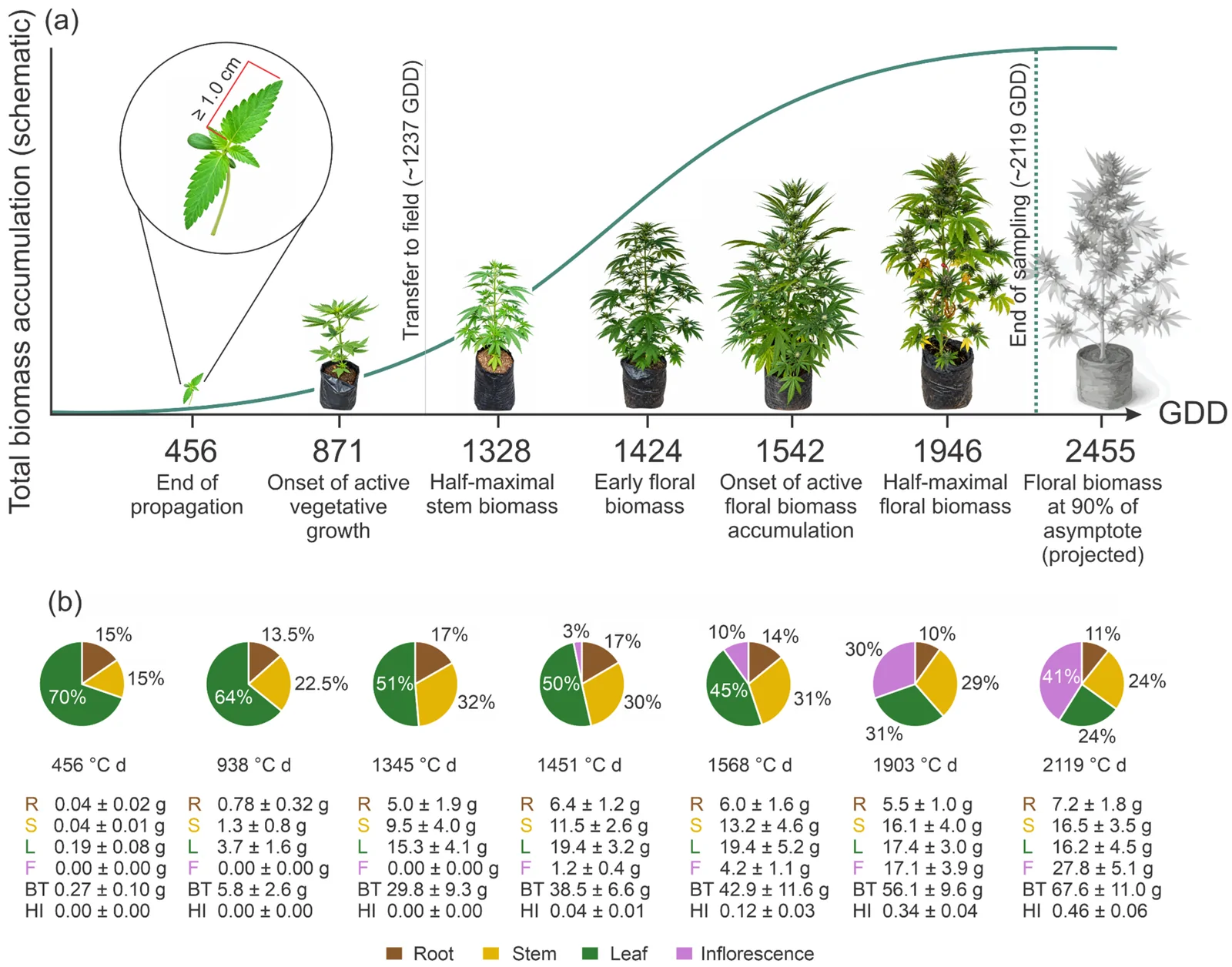
Biomass thresholds and dry-biomass partitioning in three S_1_ progenies of *Cannabis sativa* L. (**a**) Modeled total-biomass curve with developmental thresholds (Table 1), propagation endpoint (456 GDD), field transfer (1237 GDD), and final sampling (2119 GDD). Plant images illustrate developmental stages; the gray plant is AI-generated and represents a projected threshold, not confirmed maturity. Inset: unfolded leaf criterion (≥1.0 cm) [24,29]. Image spacing is not proportional to GDD. (**b**) Dry-biomass partitioning at seven sampling dates. R, root; S, stem; L, leaf; F, inflorescence; BT, total biomass; HI, harvest index. Values are means ± SD (*n* = 15). Inflorescences at 1345 GDD were not weighable. Additional photographs: Figure A1.

The fitted models identified operational biomass milestones for three S_1_ progenies (DOC, PW, and SCH, derived from Doctor, Purple Wreck, and Schanti, respectively) (Table 1, Figure 2). Thresholds up to the onset of active vegetative growth were reached under the constant 20-h photoperiod, primarily reflecting thermal accumulation and ontogeny. Model-derived thresholds from half-maximal stem biomass through half-maximal floral biomass were estimated to occur after transfer to natural short days on 5 September 2024. Because this transition also changed other environmental conditions (Section 4.2), these thresholds represent reference values specific to the management sequence rather than intrinsic thermal requirements for floral induction (Section 3.5).

Development was divided into six phases using selected model-derived thresholds and management-defined boundaries (Appendix A, Table A1), while Table 1 presents all model-derived thresholds. Propagation ended at approximately 456 GDD, when seedlings had developed a first true leaf approximately 1 cm in length and were transferred to cultivation bags. This transition represented a management-defined boundary rather than a model-derived threshold.

The subsequent model-derived thresholds were: early biomass accumulation (~582 GDD), onset of active vegetative growth (~871 GDD), half-maximal stem biomass (~1328 GDD), early floral biomass (~1424 GDD), onset of active floral biomass accumulation (~1542 GDD), and half-maximal floral biomass (~1946 GDD). Two additional thresholds were projected beyond the sampled window: harvest index at 95% of its asymptote (~2262 GDD) and floral biomass at 90% of its asymptote (~2455 GDD).

Solitary axillary flowers (codes 2001–2201 of the decimal scale [29]) were observed between 938 and 1072 GDD under the 20-h photoperiod, before any floral biomass was measurable. Condensed inflorescences appeared only after transfer to natural short days, from 1345 GDD, but were too small to be weighed; floral biomass was first measurable at 1451 GDD.

Uncertainty generally increased for later developmental thresholds, as indicated by the widening of the 95% confidence intervals (Table 1). For example, the confidence interval spanned 119 GDD for Purple Wreck (PW) at the onset of active floral biomass accumulation, whereas it spanned 1237 GDD for Schanti (SCH) at the projected threshold corresponding to 90% of maximum floral biomass accumulation. For DOC, the floral-biomass asymptote reached the upper fitting bound in 17% of bootstrap resamples; raising this bound from 10 to 100 times the largest observed value did not change the lower confidence limits of the floral thresholds, but increased the upper confidence limit for half-maximal floral biomass from 3233 to 4783 GDD, indicating that the upper confidence limits reflect the upper bound imposed on the asymptote d rather than the observed data.

Allowing *e* to differ among progenies did not significantly improve any of the six biomass models under either test. The asymptote *d* differed significantly for stems, leaves, and roots under robust inference (Holm-adjusted *p* ≤ 0.002). For total, above-ground, and floral biomass, F tests were significant (Holm-adjusted *p* = 0.028–0.041), but robust Wald tests were not (Holm-adjusted *p* = 0.11–0.21). These results do not establish equality of asymptotes. For total biomass, progeny estimates of *e* differed by only 47 GDD (1421–1468). However, the bootstrap 90% confidence intervals for pairwise differences were wide (DOC − PW, −187 to 126 GDD; DOC − SCH, −225 to 178 GDD; PW − SCH, −175 to 196 GDD), precluding conclusions about equivalence among progenies. All progenies accumulated approximately 1237 GDD before transfer. 

Under the alternative temperature-processing scenarios described in Section 4.5, accumulated GDD at the final sampling ranged from 1916 to 2035 °C d, compared with 2119 °C d in the original series. Milestones within the observed range shifted downward by up to 10% (maximum reduction of 188 °C d for half-maximal floral biomass), whereas projected milestones shifted by up to 13%. Importantly, their chronological order remained unchanged, and F tests for progeny-specific half-maximum parameters remained non-significant (total biomass, *p* = 0.55–0.58; floral biomass, *p* = 0.33).

### 2.3. Biomass Accumulation and Partitioning

**Figure 3:**
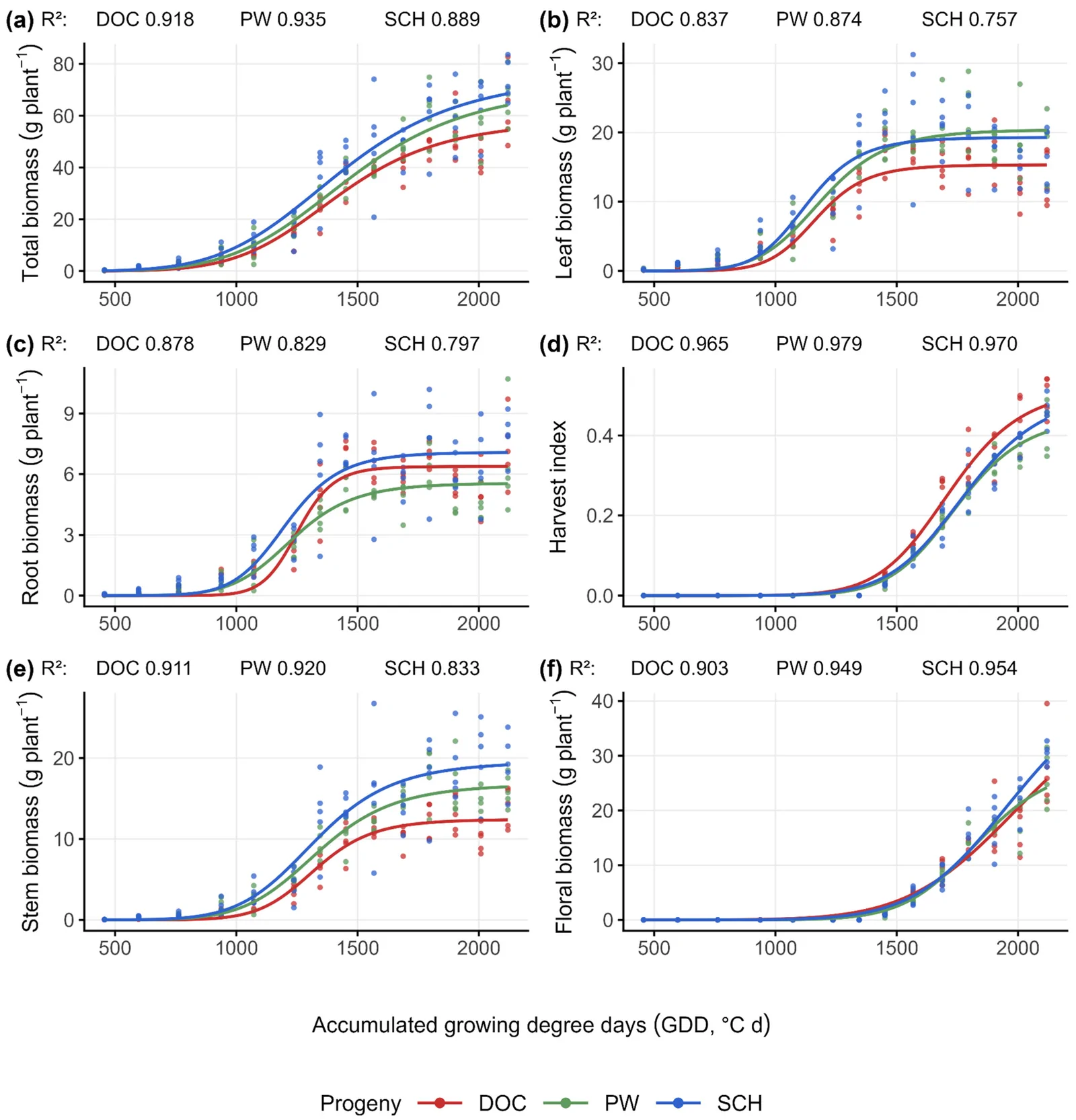
Three-parameter log-logistic models of organ-specific biomass accumulation as a function of accumulated growing degree days (GDDs) in three S_1_ progenies of *Cannabis sativa* L. Panels show (**a**) total biomass (roots included), (**b**) leaves, (**c**) roots, (**d**) harvest index, (**e**) stems and (**f**) floral biomass. Points represent observed values from weekly destructive sampling, and lines show fitted three-parameter log-logistic (LL.3) curves over the sampled range (DOC—red; PW—green; SCH—blue). Panel values indicate the R^2^ of each progeny’s fit to the individual plant observations (*n* = 70 per progeny); all fits were significant (F test, *p* < 0.001; Section 4.5). Parameter estimates and standard errors are provided in Table A3.

Three-parameter log-logistic models fitted to organ-specific biomass as a function of accumulated GDD showed sigmoidal responses across all compartments (Appendix A, Table A3; Figure 3a–f). 

Organs differed markedly in the timing of stabilization. Root biomass accumulated slowly until ~900–1000 GDD, accelerated between ~1100 and 1300 GDD, and the fitted curve approached a plateau early, at ~1500–1600 GDD, with SCH and DOC reaching higher asymptotes than PW (Figure 3c). Stem biomass remained low until ~1000 GDD, increased most rapidly between ~1200 and 1600 GDD, and the fitted curve approached a plateau between ~1900 and 2100 GDD, with SCH attaining the greatest structural mass (Figure 3e). Leaf biomass increased most rapidly between ~1000 and 1500 GDD and stabilized from ~1500–1700 GDD, with PW and SCH reaching higher asymptotes than DOC (Figure 3b).

In contrast, floral biomass showed a pronounced lag, remaining near zero until ~1400–1500 GDD, before increasing sharply between ~1600 and 2100 GDD; unlike the vegetative organs, it continued to accumulate at the end of sampling. The estimated floral-biomass asymptote was lower for PW than for DOC and SCH, whose asymptotes were less precisely estimated, although floral asymptotes did not differ significantly among progenies under heteroscedasticity-consistent inference (Figure 3f; Table A2 and Table A3). 

**Figure 4:**
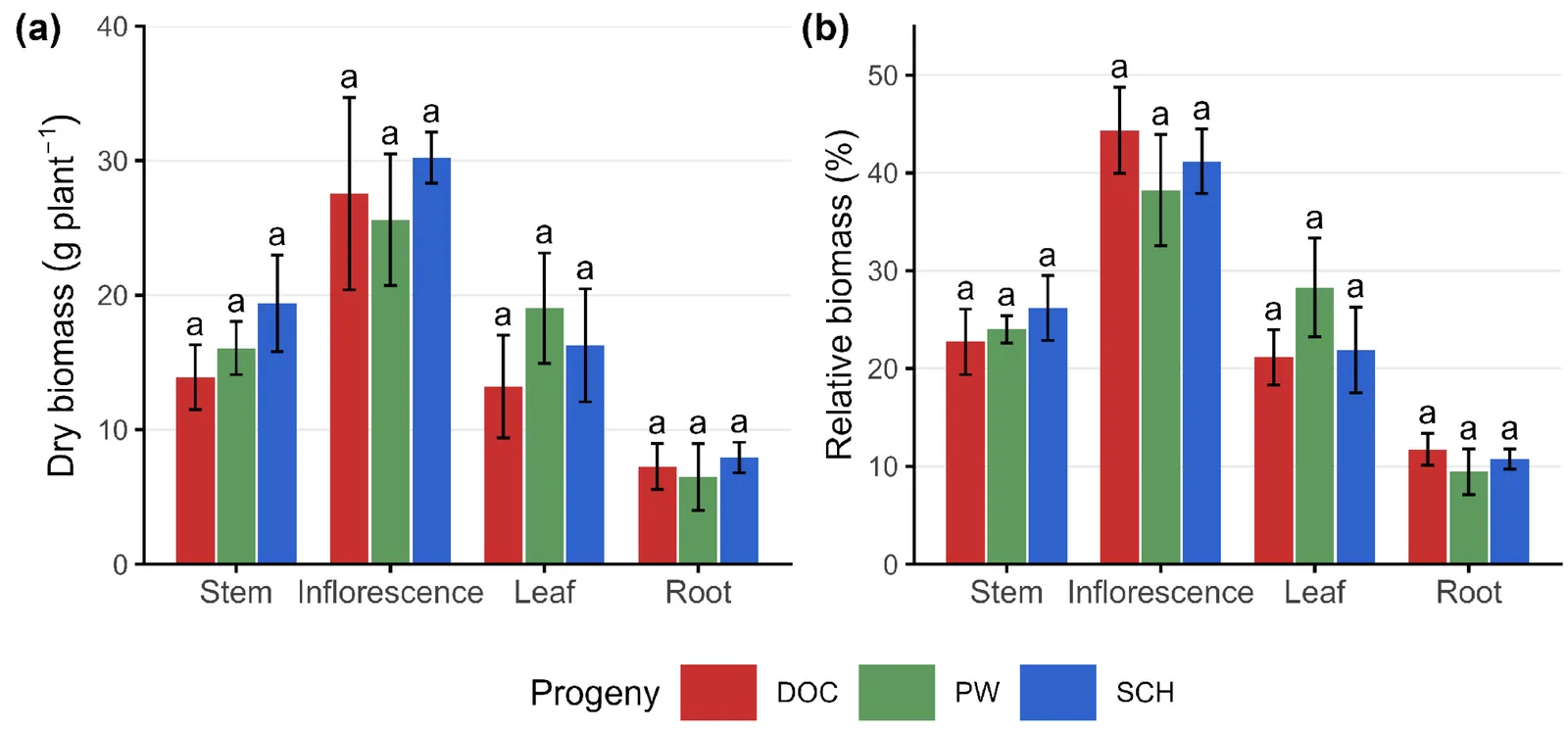
Absolute (**a**) and relative (**b**) organ dry biomass at week 14 in three S_1_ progenies of *Cannabis sativa* L. Relative biomass includes roots. Bars represent means ± SD (*n* = 5 per progeny). One-way ANOVA with Holm adjustment across organs revealed no significant differences among progenies (*p* > 0.05); Tukey’s HSD was therefore not applied, and all bars share the letter ‘a’.

Harvest index (the ratio of floral to above-ground biomass) rose steadily once floral biomass became measurable, increasing sharply between ~1600 and ~1900 GDD before gradually decelerating toward the end of the cycle (Figure 3d). The fitted curve approached but did not reach a plateau within the sampled window, with the model projecting 95% of the asymptote at ~2262 GDD. DOC maintained the highest harvest index throughout the reproductive phase (0.50 at the final sampling, compared with 0.46 in SCH and 0.42 in PW), indicating the greatest proportional allocation to reproductive biomass, whereas floral biomass at the final harvest did not differ significantly among progenies (Figure 4a).

Biomass partitioning shifted progressively throughout the developmental cycle (Figure 2b). Leaves represented 70% of whole-plant dry biomass at the end of propagation and declined monotonically to 24% by the final sampling (2119 GDD), whereas stem allocation increased from 15% to 30% by ~1345 GDD, remained at 28–32% until ~1900 GDD, and declined to 24% at the final sampling. Root allocation remained between 10% and 17% throughout. Inflorescence biomass was not detectable through 1345 GDD, but its contribution to total biomass subsequently increased from 3% at 1451 GDD to 41% at the final sampling.

Across the reproductive-phase samplings (weeks 8–14; *n* = 35 per progeny), harvest index varied significantly among progenies and sampling weeks (progeny F(2, 84) = 19.2, *p* < 0.001; sampling week F(6, 84) = 220.8, *p* < 0.001), with no significant interaction between them (F(12, 84) = 0.82, *p* = 0.63). DOC exhibited a significantly higher harvest index than both PW and SCH (Tukey, *p* < 0.001 in both cases), whereas the latter two did not differ significantly (*p* = 0.40).

At the final harvest (week 14, ~2119 GDD), floral biomass constituted the largest compartment in all progenies, averaging ~28 g plant^−1^ in DOC, ~26 g in PW and ~30 g in SCH, compared with 14–19 g for stems, 13–19 g for leaves and 6–8 g for roots (Figure 4). Floral biomass accounted for 44% of whole-plant dry mass in DOC, 41% in SCH and 38% in PW, compared with 23–26% for stems, 21–28% for leaves and 9–12% for roots (Figure 4b). At week 14, neither organ biomass nor biomass allocation differed significantly among progenies after multiple-comparison correction (*n* = 5 per progeny); prior to adjustment, nominally significant differences were observed only in stem mass (DOC < SCH) and leaf fraction (DOC < PW).

### 2.4. Morphometric Associations with Total and Floral Biomass

**Figure 5:**
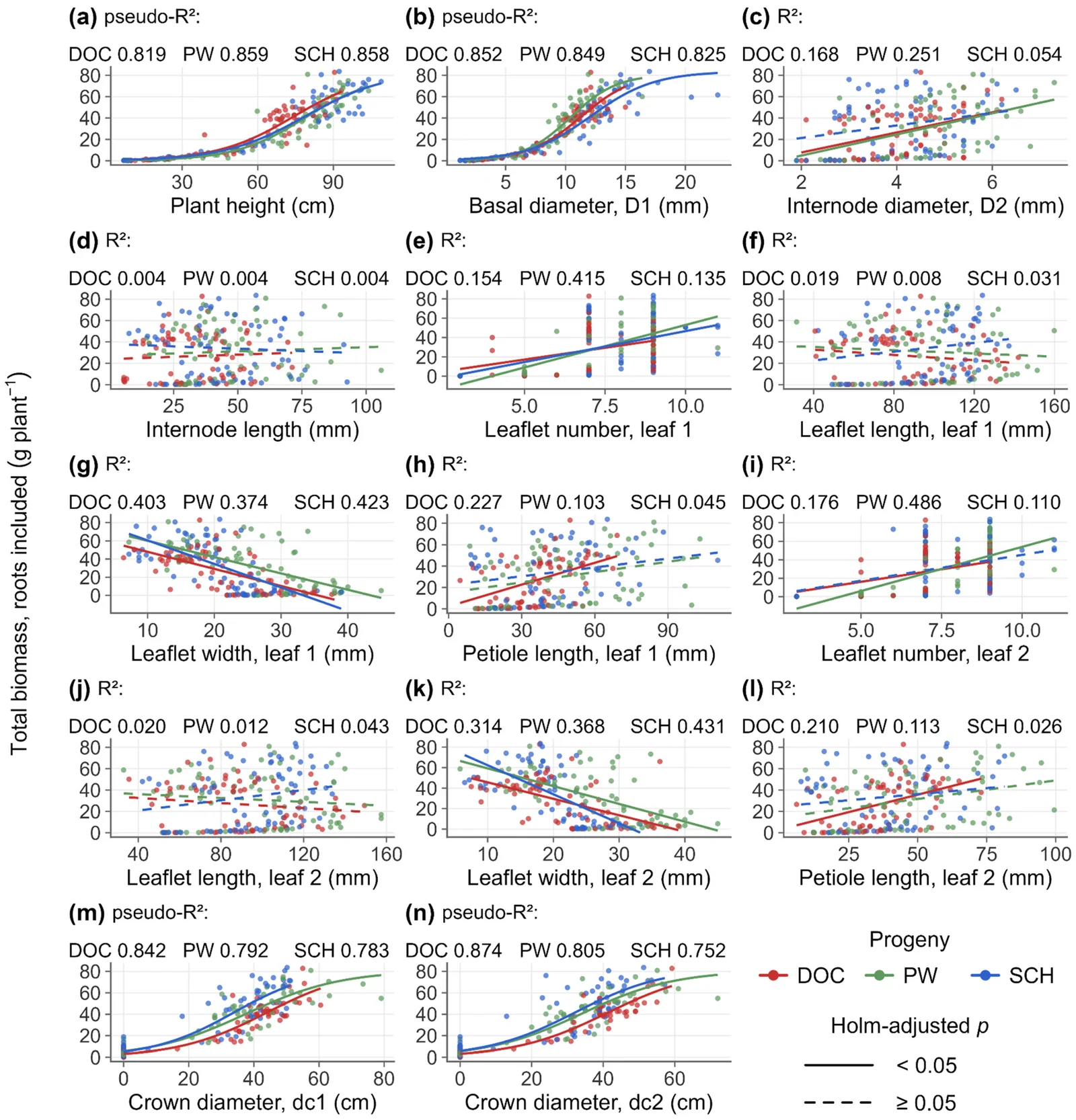
Relationships between total dry biomass (including roots) and morphometric descriptors in three S_1_ progenies of *Cannabis sativa* L. (DOC, red; PW, green; SCH, blue). Panels show relationships with: (**a**) plant height (cm); (**b**) basal diameter, D1 (mm); (**c**) internode diameter, D2 (mm); (**d**) internode length (mm); (**e**) leaflet number, leaf 1; (**f**) leaflet length, leaf 1 (mm); (**g**) leaflet width, leaf 1 (mm); (**h**) petiole length, leaf 1 (mm); (**i**) leaflet number, leaf 2; (**j**) leaflet length, leaf 2 (mm); (**k**) leaflet width, leaf 2 (mm); (**l**) petiole length, leaf 2 (mm); (**m**) crown diameter, dc1 (cm); and (**n**) crown diameter, dc2 (cm). Points represent individual plants (*n* = 70 per progeny). Lines show fitted logistic models in panels (**a**,**b**,**m**,**n**), and linear models in panels (**c**–**l**). Solid lines indicate Holm-adjusted *p* < 0.05; dashed lines indicate Holm-adjusted *p* ≥ 0.05. Panel values indicate R^2^ for linear models or deviance-based pseudo-R^2^ for logistic models. Models describe relationships across sampling dates; parameter estimates and *p*-values are provided in Table A4.

**Figure 6:**
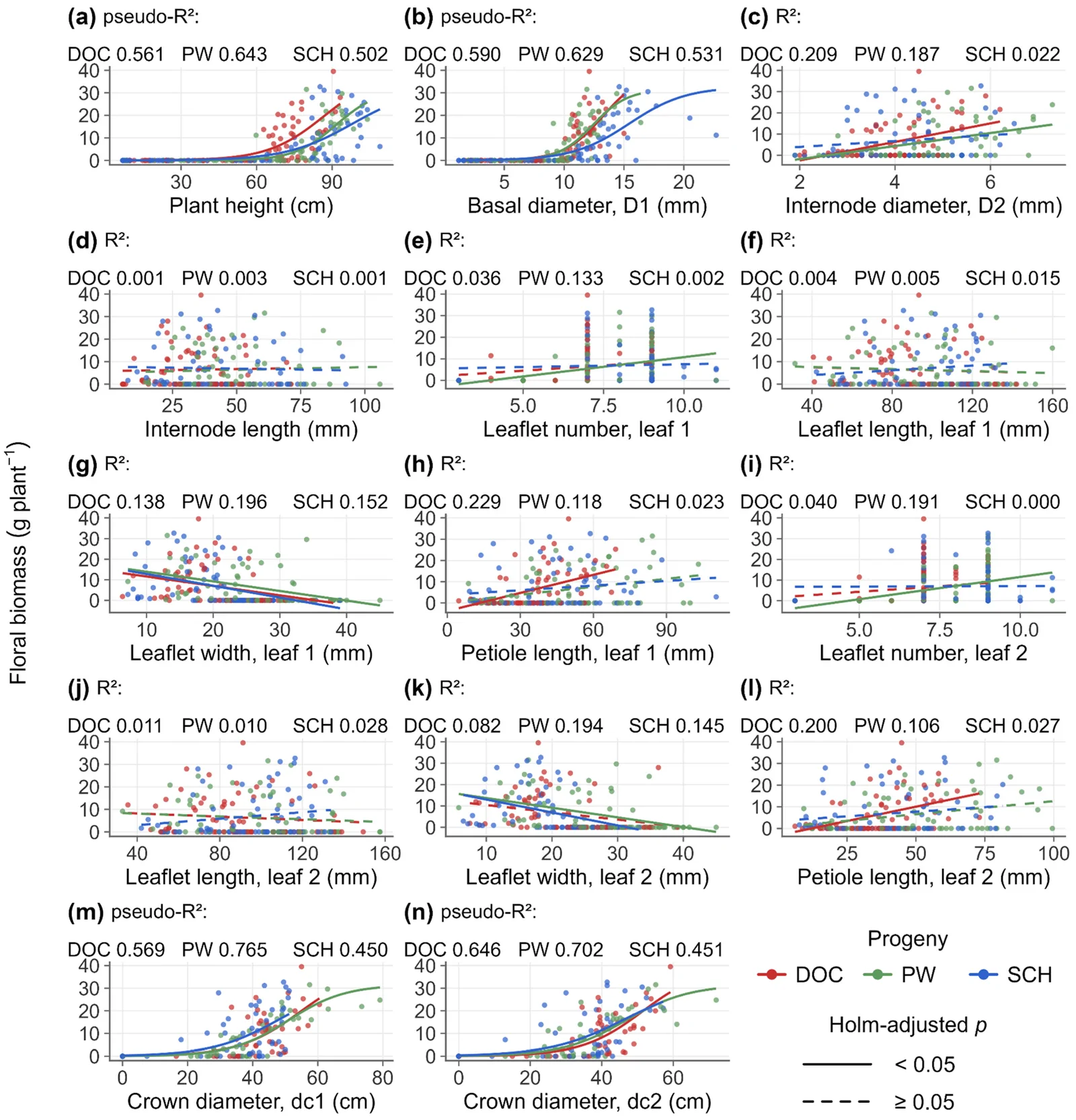
Relationships between floral dry biomass and morphometric descriptors in three S_1_ progenies of *Cannabis sativa* L. (DOC, red; PW, green; SCH, blue). Panels show relationships with: (**a**) plant height (cm); (**b**) basal diameter, D1 (mm); (**c**) internode diameter, D2 (mm); (**d**) internode length (mm); (**e**) leaflet number, leaf 1; (**f**) leaflet length, leaf 1 (mm); (**g**) leaflet width, leaf 1 (mm); (**h**) petiole length, leaf 1 (mm); (**i**) leaflet number, leaf 2; (**j**) leaflet length, leaf 2 (mm); (**k**) leaflet width, leaf 2 (mm); (**l**) petiole length, leaf 2 (mm); (**m**) crown diameter, dc1 (cm); and (**n**) crown diameter, dc2 (cm). Points represent individual plants (*n* = 70 per progeny). Lines show fitted logistic models in panels (**a**,**b**,**m**,**n**), and linear models in panels (**c**–**l**). Solid lines indicate Holm-adjusted *p* < 0.05; dashed lines indicate Holm-adjusted *p* ≥ 0.05. Panel values indicate R^2^ for linear models or deviance-based pseudo-R^2^ for logistic models. Models describe relationships across sampling dates; parameter estimates and *p*-values are provided in Table A4. GDD-adjusted analysis of four descriptors is presented in Section 2.4.

Descriptor-based models related each morphometric trait to total or floral dry biomass (logistic models for height, D1, dc1 and dc2; linear models for the remaining descriptors; Figure 5 and Figure 6; Section 4.5). 

Plant height and basal stem diameter were strongly associated with total biomass (pseudo-R^2^ ≈ 0.82–0.86 across progenies; all *p* ≤ 0.001), as was crown diameter (pseudo-R^2^ ≈ 0.75–0.87). Associations with leaflet and petiole traits varied among progenies (R^2^ up to 0.49 for leaflet number and 0.43 for leaflet width), whereas internode length showed negligible associations with either biomass response (R^2^ ≤ 0.004; Figure 5). For floral biomass, associations were weaker (pseudo-R^2^ ≈ 0.50–0.64 for height and basal stem diameter), and the best-performing descriptor differed among progenies: crown diameter ranked first in PW (dc1, pseudo-R^2^ = 0.77) and DOC (dc2, pseudo-R^2^ = 0.65), outperforming height and basal stem diameter in both, whereas in SCH crown diameter showed lower explanatory power (pseudo-R^2^ ≈ 0.45), and basal stem diameter ranked first (pseudo-R^2^ = 0.53). Across progenies, crown diameter showed the highest mean pseudo-R^2^ for floral biomass (0.60), while plant height ranked lowest among the logistic descriptors (0.57), reversing their ranking for total biomass (Figure 6). Parameter estimates for all descriptor models (intercept, coefficient, *p*-value, and R^2^ or pseudo-R^2^) for total and floral biomass are provided in Table A4 (Appendix A).

Within the reproductive window (weeks 8–14; *n* = 105), the model with GDD alone explained 86.5% of the variation in floral biomass (86.9% with progeny included as a factor; F = 1.72, *p* = 0.19). Adding individual morphometric descriptors increased explanatory power by at most 0.6 percentage points, with the largest gain for crown diameter (*p =* 0.028), followed by basal stem diameter, plant height and branch number (*p* = 0.07, 0.14 and 0.34, respectively). However, none remained significant after Holm correction for the four comparisons (smallest adjusted *p* = 0.11).

Under leave-one-week-out cross-validation, GDD alone yielded R^2^ = 0.833 (RMSE = 3.72 g per plant), compared with R^2^ = 0.844 (RMSE = 3.59 g) when crown diameter was included. Within individual sampling weeks (weeks 8–14), mean correlations with floral biomass were 0.31 for crown diameter (range: −0.04 to 0.64), 0.26 for basal stem diameter, 0.17 for branch number and 0.09 for plant height. For total biomass, plant height had the highest mean within-week correlation (0.57), compared with 0.26 for crown diameter.

## 3. Discussion

### 3.1. Phenological Progression Under the Imposed Photoperiod Sequence

Earlier hemp models describe phenological development as a function of temperature and photoperiod [15,16]. In the present experiment, all three progenies experienced the same 20-h photoperiod before being transferred to the field on the same date. Their subsequent biomass trajectories therefore reflect development following a shared management transition. Allowing progeny-specific half-maximum parameters did not significantly improve model fit (Section 2.2). This result supports reporting the fitted timing estimates for these progenies under the experimental conditions, but does not establish equivalent thermal requirements.

Floral development involves two distinct regulatory stages, with overlapping vegetative growth and reproductive differentiation. Solitary axillary flowers are initiated at the juvenile-to-adult transition through age-dependent, photoperiod-independent signals and can differentiate even under long days [30,31]. Short days then trigger two successive phases of shoot-apex development: internodes and the main stem first elongate rapidly, followed by the cessation of elongation in newly formed internodes and the formation of condensed inflorescences through extensive apical branching and shortening of internodes and petioles [30,32]. This second step requires short days in photoperiod-sensitive genotypes such as those evaluated here, whereas autoflowering types flower independently of daylength [33,34].

The progenies evaluated here followed this sequence. Flower primordia and solitary flowers developed under the 20-h photoperiod, whereas condensed inflorescences and measurable floral biomass appeared only after transfer to natural short days (Section 2.2). Trichome development and maturation depend on plant age and genotype [35] but were not assessed in this experiment. The projected 90% threshold of the fitted floral-biomass asymptote therefore represents late biomass accumulation rather than an observed transition to physiological or medicinal maturity.

Point estimates were similar among progenies for half-maximal stem biomass (1325–1337 GDD), whereas later thresholds, especially the projected 90% floral-biomass threshold, were less precise and, for DOC, partly dependent on the fitting bound (Section 2.2; Table 1). Thus, comparisons at later developmental stages remain uncertain, and similar point estimates do not establish equivalence.

At the study latitude (22°25′ S), sunrise-to-sunset daylength increased from approximately 11.1 h in early August to 12.9 h in early November. The minimum critical photoperiod reported among 27 hemp cultivars was 13 h 45 min to 14 h [36], although photoperiodic responses under tropical conditions vary with genotype and temperature [19]. Natural daylength at the site remained below this reported range, and supplemental lighting was used to maintain vegetative growth. The subsequent increase in floral biomass is consistent with the photoperiodic response of the evaluated material, but its rate cannot be attributed to temperature alone, as other environmental conditions also changed at transfer (Section 4.2).

These findings characterize the sequence of vegetative growth and floral biomass accumulation under the evaluated management regime. Assessing the consistency of these thermal thresholds across environments would require replicated planting or transfer dates across progenies. Photoperiod treatments under comparable microclimatic conditions could further distinguish photoperiodic effects from environmental changes associated with transfer.

### 3.2. Thermal-Time–Based Modeling of Biomass Accumulation

The biomass accumulation patterns observed here are consistent with those reported in a greenhouse study of medicinal *Cannabis sativa* L. that used growing degree days (GDDs) as an explanatory variable, fitted logistic functions to organ-level biomass during flowering (R^2^ = 0.89–0.91), and applied phase-specific base temperatures (1.9 °C vegetative, 11.3 °C generative) [5]. The resulting logistic trajectory comprises three segments corresponding to the flowering phases identified here, although the third phase extends beyond the sampling period and remains a projection. Their correspondence with the decimal code for hemp growth stages [29] and GDD thresholds is presented in Table A1 (Appendix A). In the same study, inflorescence and CBD yields increased linearly with GDD, while harvest index did not differ significantly among growing media (approximately 0.56).

Changing the base temperature (*Tb*) from 1.9 °C to 11.3 °C at transfer alters daily GDD accumulation. For example, a day with *Tmax* = 26 °C and *Tmin* = 16 °C contributes 19.1 GDD at the first base temperature and 9.7 GDD at the second. Although cumulative GDD remains additive, it combines two temperature-response conventions. In the original hemp model [15], phase-specific temperatures were used in a nonlinear response to hourly temperature; their application to a linear GDD sum, as here and in an earlier medicinal-cannabis study [5], represents an operational approximation. Consequently, thermal thresholds must be interpreted according to the specified base-temperature switching rule, and comparisons with single-*Tb* studies require recalculation using a common scale.

In the present study, three-parameter log-logistic (LL.3) models described GDD-dependent organ biomass accumulation, with R^2^ values ranging from 0.76 to 0.98. Evidence from studies examining other environmental factors provides additional context for interpreting these growth patterns. In a study of three medicinal cannabis cultivars grown under contrasting light spectra [37], each cultivar maintained its characteristic morphology and growth pattern, with the main response being stem elongation under a low red/far-red ratio (shade avoidance). Although this study examined light rather than temperature, it suggests that cultivar-specific growth patterns may persist under different growing conditions. Process-based modeling provides another comparison. An adapted CROPGRO framework [38] simulated above-ground biomass (Willmott’s d = 0.91) and flowering time (errors of +4 to −5 days) in fiber and seed hemp. However, inflorescence biomass was not modeled, and agreement with observations does not validate the present models, which were assessed only using the fitting data (Section 3.5).

### 3.3. Biomass Partitioning and Harvest Index Dynamics

Harvest-index values reported for medicinal *Cannabis sativa* L. vary with plant material, cultivation conditions, and the definition of the harvested fraction. In high-CBD cannabis, the harvest index ranged from 0.60 to 0.65 and responded significantly to fertilizer restriction and shoot-number manipulation [20], whereas another fertilization study reported no significant treatment effects [39]. Water deficit can also alter reproductive allocation, with a decline in harvest index reported for CBD-dominant material under drought [40]. Although no deficit treatment was imposed and plants were irrigated to drainage, plant water status was not measured.

DOC exhibited a higher harvest index without a significant advantage in final floral biomass (Section 2.3), indicating greater proportional reproductive allocation rather than higher absolute floral yield. This distinction supports evaluating absolute floral biomass alongside harvest index when comparing plant material for medicinal production.

Source–sink relationships may contribute to these patterns. Floral biomass depends on carbon supply and allocation to reproductive growth, whereas harvest index also reflects biomass retained in stems and leaves. Variation in canopy architecture, photosynthetic activity, reproductive sink strength, nutrient allocation, or leaf retention could therefore produce different biomass and harvest-index trajectories despite similar fitted timing. Differences in sink strength and nutrient allocation have been documented in drug-type chemovars [23], but these mechanisms were not assessed here and cannot be identified as causes of the observed progeny differences.

Comparisons across studies also require consistent definitions of floral tissue. Harvest-index values of 0.57–0.60 were reported for two THC-dominant cultivars with sugar leaves included in their apical inflorescences [41], whereas sugar leaves were assigned to the leaf compartment in the present study. A medicinal-genotype panel reported values of 0.10–0.30 for trimmed and refined inflorescences [26]. These differences in tissue classification affect the numerator and may influence comparisons even when biomass allocation is similar. Reported harvest-index values should therefore specify both the floral-biomass definition and the denominator used.

Harvest index increased during reproductive development, with the largest observed weekly increments between approximately 1450 and 1800 GDD and smaller gains thereafter. No plateau was observed before the final sampling (~2119 GDD). Continued reproductive growth has also been reported in high-CBD cannabis [42], while canopy senescence during late flowering [43] may influence harvest index through changes in vegetative biomass. As senescence was not quantified, its contribution remains uncertain. Similar thermal accumulation across seasons can also result in different reproductive yields and allocation patterns [44]. Harvest index should therefore be interpreted alongside floral biomass and product maturity; neither its increase nor the projected 95% asymptotic threshold establishes an optimal harvest date.

### 3.4. Potential of Morphometric Traits for Non-Destructive Biomass Estimation

The strongest morphometric association varied with the biomass compartment. Plant height and basal stem diameter were strongly associated with total biomass across sampling dates, whereas crown diameter showed the highest floral-biomass pseudo-R^2^ in two of the three progenies (Section 2.4). These patterns are biologically plausible: height and stem diameter reflect structural growth, whereas crown width may reflect the distribution of inflorescence-bearing lateral branches. As these traits can be measured on intact plants, they warrant further evaluation for non-destructive biomass estimation. However, their predictive performance requires independent validation.

Candidate equations should be validated using independent plants or growing cycles spanning the intended range of plant sizes and developmental stages, while accounting for progeny differences. Their use for selection at a common developmental stage requires separate validation. Direct biomass measurements remain necessary for calibration and validation; the limited gain beyond GDD does not imply that morphometric traits are unsuitable for biomass estimation.

Studies in other populations reinforce the need to validate proxies in the target material. In field-grown hemp, basal stem diameter was the best single criterion for floral biomass, with canopy kite area also among the leading predictors [25]. In a medicinal-genotype panel, vegetative measurements yielded a floral-biomass prediction equation, whereas floral bud dry weight was uncorrelated with days to maturation [26]. These studies support testing architecture-based proxies but do not validate their ranking or performance in the present progenies.

The UPOV guidelines [24] address distinctness, uniformity, and stability, rather than yield prediction; although TG/276/2 introduced separate variety types for horticultural cultivation in 2025, plant height remains its only whole-plant size characteristic. Combining floral biomass with cannabinoid concentration could allow breeding programs to assess per-plant cannabinoid yield alongside architectural traits. Trichome density alone does not consistently predict cannabinoid concentration across genotypes [45]. Because cannabinoids and trichome development were not measured here, these associations apply only to biomass, not resin potency or medicinal product quality.

### 3.5. Scope of Application and Further Validation

The LL.3 models provided a close fit to the observed biomass and harvest-index trajectories, supporting thermal time as a useful framework for describing growth under the conditions evaluated. Together with the morphometric relationships, these results provide a basis for developing non-destructive biomass-estimation tools and organizing crop observations and sampling. Their potential for adaptation to other production settings merits evaluation using independent data.

The numerical GDD thresholds reported here are specific to the present calibration, which encompasses one site, one season and three segregating S_1_ families grown under a defined management sequence, in 19 L bags that may restrict root growth relative to in-ground cultivation, and under a single irrigation regime. Family-level differences should therefore be distinguished from cultivar- or chemotype-wide effects. The greenhouse-to-field transfer changed photoperiod and other environmental conditions simultaneously, so post-transfer GDD thresholds represent operational references for that management sequence rather than isolated temperature requirements. The available leave-one-week-out assessment concerns the complementary linear GDD models within this experiment.

Temperature measurement is an important consideration for further calibration. The logger shelter, which had limited ventilation, may have elevated maximum readings, and applying the 40 °C ceiling limited extreme contributions without correcting the underlying measurement uncertainty. Nevertheless, the sensitivity analyses (Section 2.2) preserved the order of the biomass milestones and the non-significant tests of progeny-specific half-maximum parameters across the scenarios examined. These results support the consistency of the observed sequence across the tested scenarios, while indicating that numerical thresholds depend on the temperature record and calculation procedure; the scenarios do not bound the possible measurement error.

Further applications should evaluate the equations across planting dates, plant materials and production systems, using air temperature measured in a ventilated radiation shield and a consistent GDD calculation. Such calibration is particularly relevant in protected cultivation, where temperature, vapor pressure deficit and radiation jointly influence crop performance [46]. Additional observations are needed to assess the projected thresholds at 90% of the floral-biomass asymptote and 95% of the harvest-index asymptote, which lie beyond the sampled period. Within these boundaries, biomass trajectories can support crop monitoring and preparation for harvest, while harvest decisions should integrate floral biomass, which is not equivalent to commercial yield, with cannabinoid yield and composition, trichome maturity, and other product-quality measurements, none of which were determined here. 

## 4. Materials and Methods

### 4.1. Location

The experiment was conducted at a medical cannabis patient association, APEPI (Association for the Support of Research and Patients Using Medical Cannabis), located in the rural area of the municipality of Paty do Alferes, Rio de Janeiro State, Brazil (22°25′44″ S, 43°24′08″ W; 624 m above mean sea level).

In the Köppen classification, the climate of the study area is Cfa (humid subtropical), with hot, humid summers, mild winters, and well-distributed annual precipitation, typical of parts of southeastern Brazil [47]. The classification refers to long-term climatic normals. The Cfa classification reflects the elevation of the site (624 m); at 22°25′ S the site lies within the tropics, and the daylength regime is that of a tropical latitude. References to tropical conditions throughout this work denote this photoperiodic regime rather than the thermal classification.

### 4.2. Plant Material and Photoperiod Conditions

S_1_ seeds were produced by self-pollination following chemically induced sex reversal of female mother plants using 3 mM silver thiosulfate (STS), an inhibitor of ethylene action that induces viable male flowers in genetically female (XX) plants [48]. Ethylene-mediated sex reversal in *Cannabis sativa* L. involves early transcriptional reprogramming preceding floral development [49].

Three exclusively female S_1_ progenies were evaluated, each derived from a selfed clonal cultivar: Purple Wreck (THC-dominant), Doctor (CBD-dominant), and Schanti (balanced THC:CBD ratio). Cannabinoid profiles were determined in mature plants of the same S_1_ progenies from a separate experiment conducted concurrently in an adjacent area, not in the plants destructively sampled here; these data will be reported in a separate publication. 

All plant material was handled by the research team of the Active Germplasm Bank of Cannabis (AGBC) at the Federal University of Viçosa (UFV) under a judicial protection order issued in a preventive habeas corpus proceeding No. 6000670-64.2024.4.06.3823/MG, authorizing research activities within the scope established by the Brazilian Federal Judiciary.

**Figure 7:**
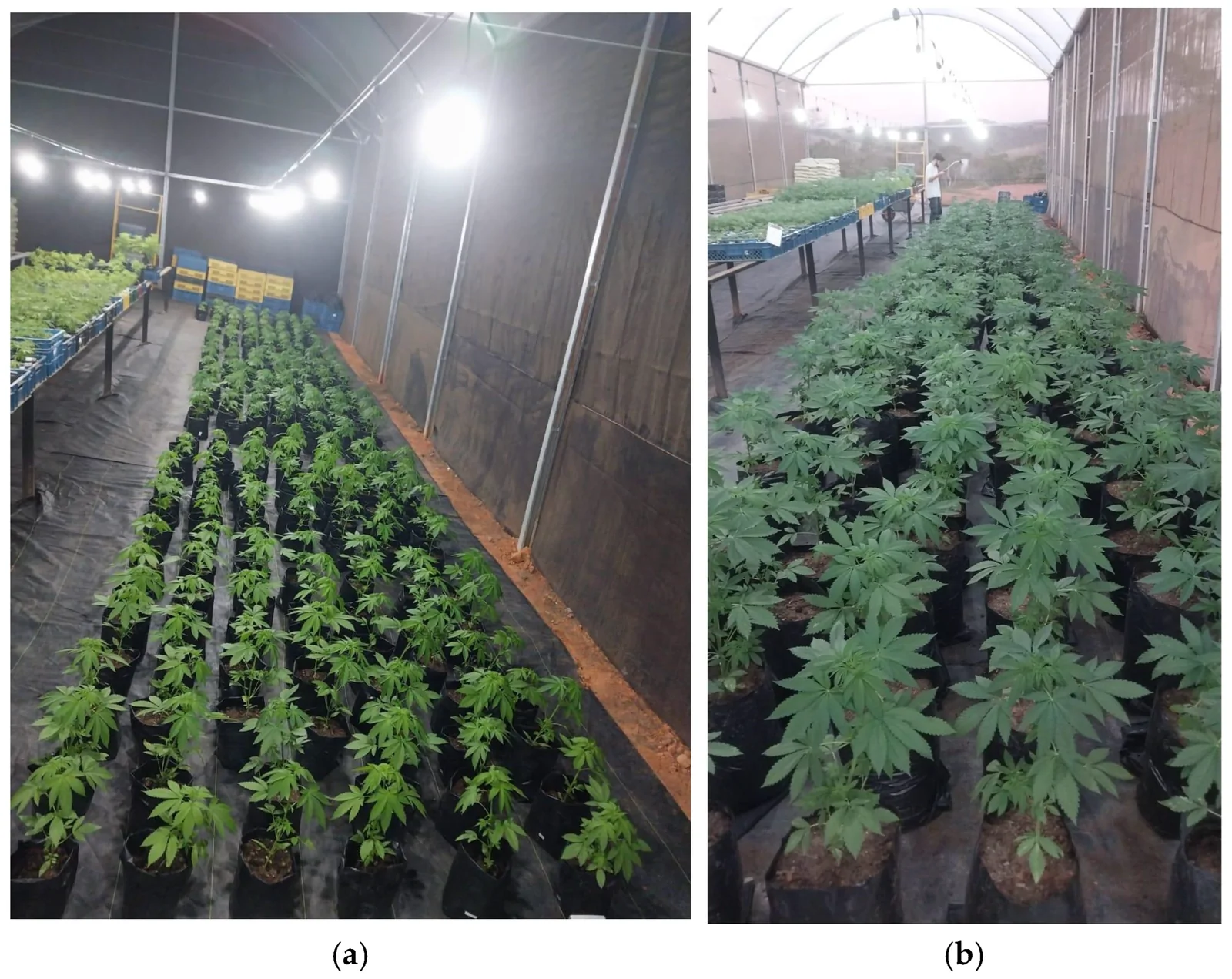
Medicinal *Cannabis sativa* L. plants during the vegetative phase grown in a greenhouse under supplemental lighting (20-h photoperiod). (**a**) Young plants in the third week of vegetative growth. (**b**) Plants in the fourth week of the vegetative stage.

The three medicinal cannabis S_1_ progenies were grown in a greenhouse under a 20-h photoperiod during vegetative growth (Figure 7). Supplemental lighting consisted of 9 W LED lamps spaced 1 m apart in a line, providing an approximate power density of 3.6 W m^−2^. Assuming a white-LED photon efficacy of 1.7 µmol s^−1^ W^−1^, the estimated nominal photon flux density was ~6 µmol m^−2^ s^−1^; canopy photon flux was not measured. The lamps extended the natural day without interrupting the night, leaving a 4-h dark period. At this estimated intensity, supplemental lighting was expected to act primarily as a photoperiodic signal, with little contribution to net photosynthetic carbon assimilation.

**Figure 8:**
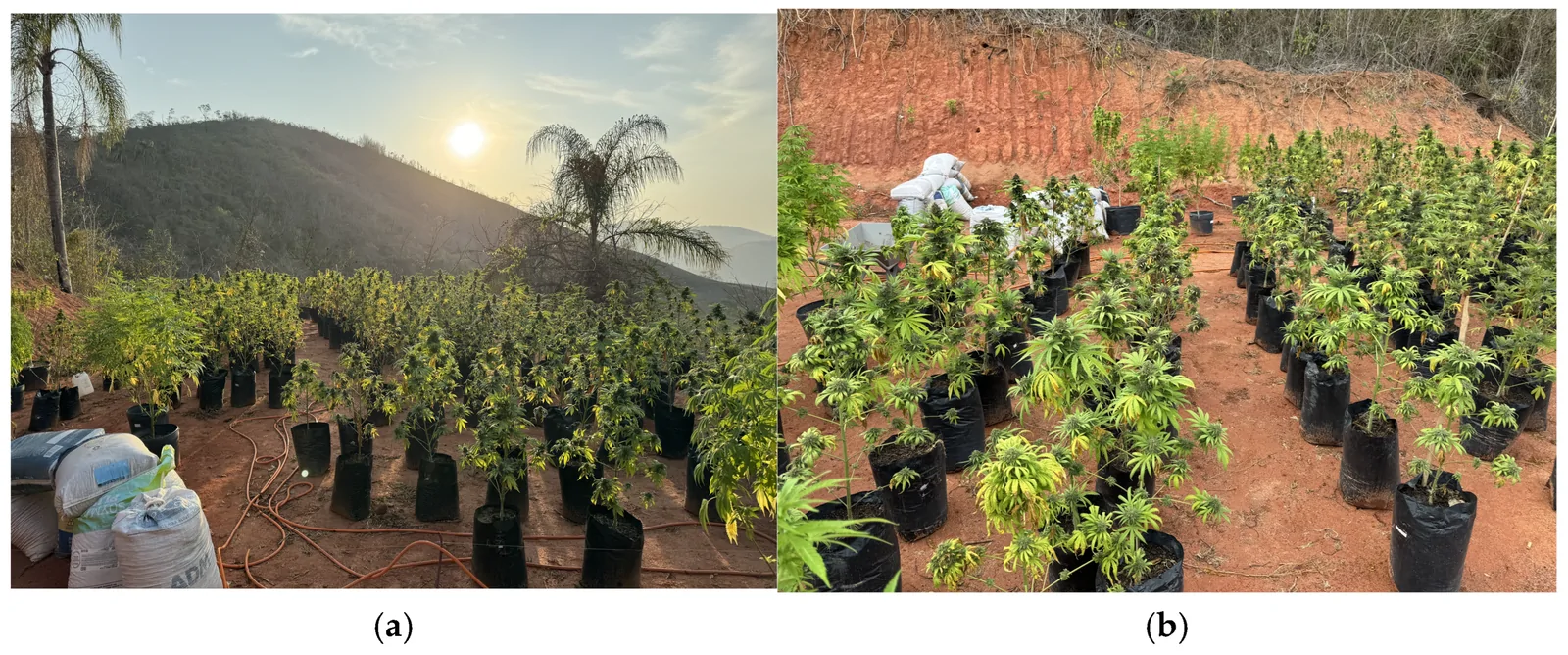
Flowering phase of medicinal cannabis cultivated under open-field conditions during the 2024 growing season. (**a**) General view of the experimental area with plants at the flowering stage. (**b**) Detailed view of the crop rows.

On 5 September 2024, all three progenies were transferred to open-field cultivation under natural photoperiod conditions, without supplemental lighting, through October 2024 (Figure 8). This transition simultaneously altered photoperiod, incident radiation, vapor pressure deficit, exposure to wind and rainfall, and thermal conditions.

### 4.3. Crop Management Practices 

Seeds were soaked in water overnight and subsequently germinated on moist germination paper. Following radicle emergence, seedlings were transplanted into 1 L pots containing an acclimatization substrate composed, by volume, of Carolina Soil (inert matrix, 70%), cured manure (10%), organic compost (10%), and earthworm humus (10%).

Later during vegetative growth, plants were transferred to 19 L cultivation bags containing a substrate composed, by volume, of earthworm humus (10%), organic compost (20%), clay soil (5%), cured manure (20%), sand (10%), raw rice husk (10%), coconut fiber (15%), and bagasse or leaf litter (10%).

Fertilization consisted of powdered formulations. During the vegetative phase, 250 g of fertilizer mixture per plant was incorporated into the substrate, comprising, by mass, thermophosphate (58%), castor cake (12%), potassium sulfate (8%), bone meal (5%), Ekosil^®^ (5%; milled phonolite, a potassic silicate rock containing 8% K_2_O; Yoorin Fertilizantes, Poços de Caldas, Brazil), cured manure (7%), and rice husk (5%). At mid-flowering, 120 g per plant was applied as topdressing, comprising potassium sulfate (40%), bone meal (30%), and rice husk (30%).

Plants were manually irrigated four times weekly until drainage occurred, with irrigation reduced to twice weekly during the final three weeks of flowering, following the routine production practice at APEPI. Field-grown plants also received natural rainfall. For phytosanitary control, matrine (Matrine^®^, Dinagro, Ribeirão Preto, Brazil; ethanolic extract of Sophora flavescens at 190.5 g L^−1^, equivalent to 2.0 g L^−1^ oxymatrine; soluble concentrate) was applied three times at five-day intervals near the end of vegetative growth. Applications followed the midpoint of the labeled concentration range for two-spotted spider mite (*Tetranychus urticae*; 0.15–0.30 L per 100 L of water), corresponding to 0.225 L per 100 L.

### 4.4. Experimental Design and Sampling Procedures

A completely randomized design was used, with plants spaced 0.7 m within rows and 1.2 m between rows. Plants were randomly assigned to greenhouse positions, and the same spatial arrangement was maintained after field transfer. At each sampling, five plants per progeny were randomly selected. Gaps left by harvested plants were filled by repositioning adjacent plants to maintain a continuous stand. No border plants were designated, and all plants with the expected photoperiodic response remained eligible for sampling. Spatial effects were not explicitly modeled; randomized positioning and sampling were intended to distribute potential positional effects across progenies and sampling dates.

Destructive sampling was conducted weekly, with five plants per S_1_ progeny (one family per parental cultivar), totaling 15 plants per sampling and 210 plants over 14 weeks. Sampling dates, accumulated GDD, and growth stages are provided in Table A5 (Appendix A). Each plant was separated into roots, stems, leaves, and inflorescences when present.

Bracts were included in the inflorescence fraction, whereas sugar leaves were classified as leaf biomass. Harvest index was calculated as the ratio of floral dry biomass to total above-ground dry biomass (flowers, stems, and leaves), excluding roots. Reported values represent means of individual plant ratios.

**Figure 9:**
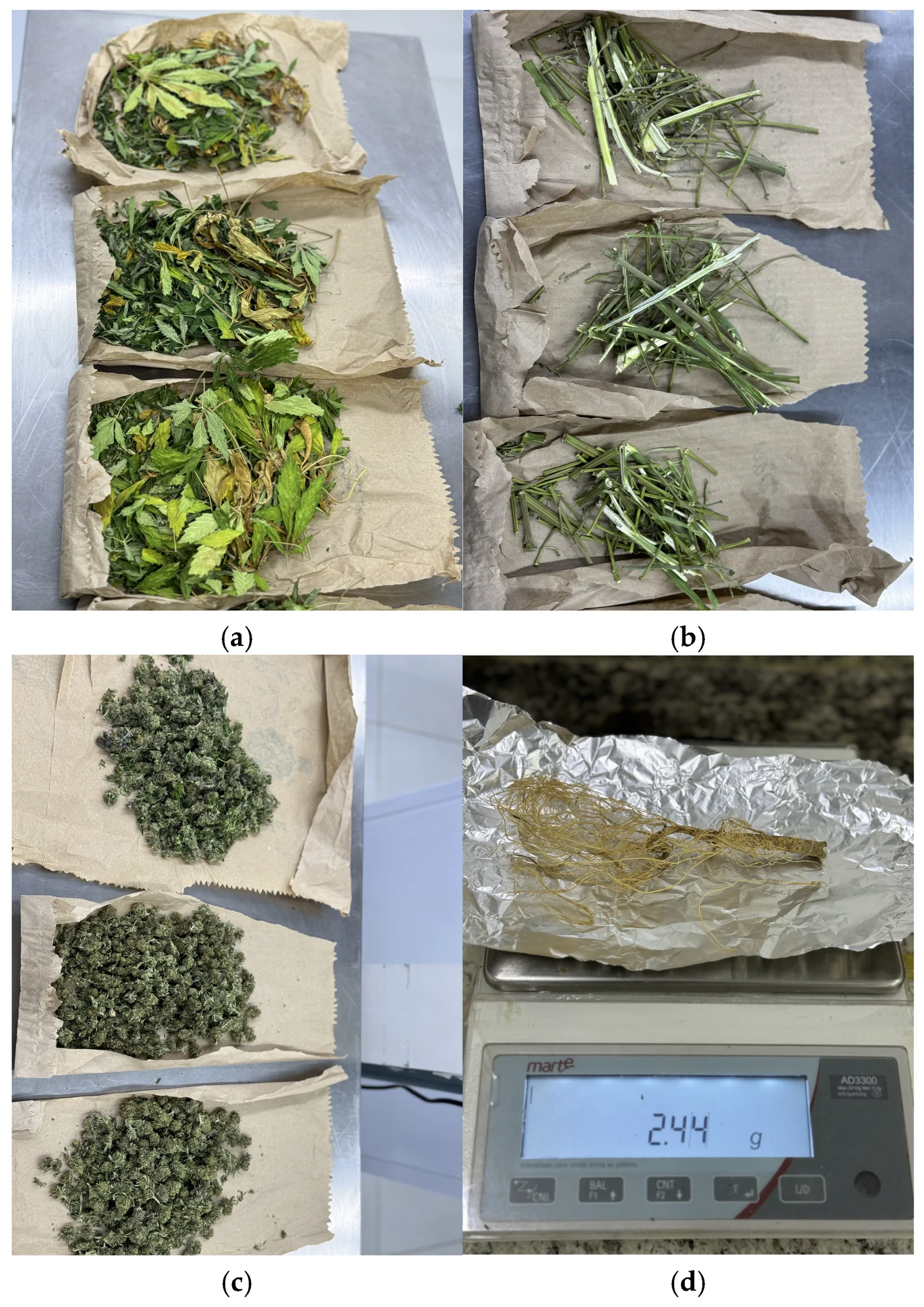
Plant organ processing and weighing for dry-biomass determination. Dried samples were separated by plant and organ: (**a**) leaves, (**b**) stems, (**c**) inflorescences, and (**d**) weighing of cleaned roots.

Each organ was placed in a separate, labeled, breathable bag and oven-dried at 65 °C under forced-air circulation until constant weight (minimum 24 h). Dry biomass was then determined by weighing (Figure 9).

Morphometric descriptors were recorded on each sampled plant before harvest. Plant height and leaf and internode traits were measured following a protocol adapted from the UPOV test guidelines for *Cannabis sativa* L. [24] for horticultural varieties propagated from feminized seed (type HF). The protocol specifies the use of fully developed leaves from the center of the plant without prescribing a specific node. Plant height was measured from soil level to the plant apex, including the inflorescence. Two fully developed leaves from the same central node (leaves 1 and 2) were assessed for leaflet number, central leaflet length and width, and petiole length. Internode length and diameter (D2) were measured on the main-stem internode immediately below that node, consistent with the TG/276/2 position for stem thickness. Three additional descriptors were recorded: basal stem diameter (D1), measured at the base of the main stem at soil level using a digital caliper; crown diameter, measured with a measuring tape as the maximum horizontal canopy width (dc1) and its perpendicular width (dc2); and branch number, defined as the number of primary branches on the main stem.

### 4.5. Statistical Analysis

Growing degree days (GDDs) were calculated using the averaging method (Method 2 in [50]), whereby daily maximum and minimum temperatures were bounded by the base and ceiling temperatures before averaging. GDD were accumulated from the onset of propagation (10 July 2024) to the day preceding each morning sampling. Daily temperatures were recorded using a Tinytag data logger (Gemini Data Loggers (UK) Ltd., Chichester, UK) from 10 July to 31 October 2024, inside the greenhouse during propagation and vegetative growth and in the field following transfer (Section 4.2). The logger was positioned approximately 1 m above ground, near the canopy, within a plastic shelter with limited ventilation and protection from direct solar exposure. Consequently, maximum readings on clear days may have overestimated air temperature. The ceiling temperature (*Tc*) of 40.0 °C limited this potential bias in calculated GDD; daily maxima reached *Tc* on 32 of 114 days.

Sensitivity to this potential bias was assessed by recalculating GDD with *Tc* reduced to 35 or 32 °C or by subtracting 2 or 4 °C from each recorded daily maximum, followed by refitting the pooled LL.3 models and nested F tests for progeny-specific half-maximum parameters described below. Data from the A637—Paty do Alferes (Avelar, RJ) meteorological station [28] provided rainfall and regional temperature references (Section 2.1, Figure 1) but were not used to calculate GDD; daily rainfall was aggregated by station day (UTC). Daily thermal time (DTT) on day t was calculated as

(1) $${\mathrm{DTT}}_{t}=\left[\frac{\min\;\left(T_{c},\max\;\left(T_{b},T_{\max,t}\right)\right)+\min\;\left(T_{c},\max\;\left(T_{b},T_{\min,t}\right)\right)}{2}\right]-T_{b}$$

where *T_max_*_,*t*_ and *T_min_*_,*t*_ are the daily maximum and minimum temperatures recorded by the logger (°C) on day *t*, each constrained to the interval between the base temperature (*T_b_*) and the ceiling temperature (*T_c_*). For each temperature, the maximum function (*max*) first applies the lower threshold, replacing values below *T_b_* with *T_b_*, whereas the minimum function (*min*) subsequently applies the upper threshold, replacing values above *T_c_* with *T_c_*. Daily thermal time (*DTT_t_*) is then calculated as the arithmetic mean of the adjusted maximum and minimum temperatures, minus the base temperature. Consequently, daily thermal time ranges from zero to *T_c_* − *T_b_* and cannot be negative.

Cardinal temperatures were adopted from a hemp phenological model [15], without estimation from the present data: *T_b_* = 1.9 °C before transfer to natural short days (5 September 2024) and 11.3 °C thereafter, with *T_c_* = 40.0 °C throughout the experiment. The change in *T_b_* was predefined by the transfer date, independently of the estimated flowering thresholds. The same base and ceiling temperatures have also been used for medicinal cannabis under greenhouse conditions [5]. Cumulative growing degree days (GDDs) were calculated by summing daily thermal time (DTT) values from the beginning of the experimental period to day i:

(2) $$\mathrm{GDDᵢ}\;=\sum_{t\;=\;1}^{i}{\mathrm{DTT}}_{t}$$

where *DTT_t_* represents the daily thermal time on day *t*. Biomass accumulation curves were modeled as a function of accumulated growing degree days (GDDs) using a three-parameter log-logistic function (LL.3), expressed as

(3) $$f\left(x\right)=\frac{d}{1+\exp\left(b\left(\log\left(x\right)-\log\left(e\right)\right)\right)},$$

where, in Equation (3), *x* is the accumulated GDD. Parameter d represents the fitted upper asymptote, e is the half-maximum parameter (ED50), corresponding to the GDD at which the curve reaches 50% of d, and b is the slope parameter. Models were fitted by nonlinear least squares separately for each S_1_ progeny (*n* = 70) and the pooled dataset (*n* = 210). Model fit was assessed by R^2^, residual analysis and visual comparison of observed and fitted values, and the overall significance of each progeny-specific fit by an F test against an intercept-only model (2 and *n* − 3 degrees of freedom). As destructive sampling precluded repeated measurements of individual plants, no plant-level random effect was required. However, plants sampled in the same week shared environmental conditions; therefore, cross-validation (described below) excluded entire sampling weeks, although this procedure does not establish residual independence in the fitted models.

Phenological thresholds were defined as the accumulated GDD at which each fitted LL.3 curve reached a specified proportion of its upper asymptote (*d*), calculated by inverting the fitted function. Early biomass accumulation and onset of active vegetative growth were defined at 0.5% and 5% of the total-biomass asymptote, respectively, and half-maximal stem biomass at 50% of the stem-biomass asymptote (parameter *e*). The distinction between early and active vegetative growth was an operational criterion, not a stage of the decimal code for hemp [29], which subdivides vegetative growth by node number. Early floral biomass, onset of active floral biomass accumulation, and half-maximal floral biomass were defined at 5%, 10%, and 50% of the floral-biomass asymptote, respectively. Additional thresholds were established at 90% of the floral-biomass asymptote and 95% of the fitted harvest-index asymptote.

Because residual variance increased with fitted biomass, the F tests of overall fit are approximate, and uncertainty was assessed using methods that accommodate heteroscedasticity. The 95% confidence intervals for thresholds (Table 1) were obtained by percentile bootstrap (10,000 resamples), resampling whole plants with replacement within each progeny × sampling-week stratum (five plants per stratum), refitting LL.3 models, and recalculating thresholds. Parameter bounds were −100 ≤ b ≤ −0.5, 0 ≤ d ≤ 10 times the maximum observed response (recalculated for each resample), and e between the minimum and 10 times the maximum GDD. Resamples reaching parameter bounds were retained, and all models converged (Table 1 note). 

For DOC floral biomass, whose asymptote frequently reached the upper bound, the same resamples were refitted with the upper bound of d increased to 20, 50 and 100 times the largest observed value, as diagnostic scenarios without biological validation. Bootstrap intervals accommodate asymmetric sampling distributions of e and the derived thresholds. Parameter standard errors (Table A3) were calculated from heteroscedasticity-consistent (HC1) covariance matrices based on the Hessian of the residual sum of squares and were not used to construct the confidence intervals in Table 1. Stratified resampling allows variance to differ among progenies and sampling weeks but does not account for potential dependence among plants sampled within the same week. With only five plants per stratum, nominal confidence interval coverage cannot be guaranteed.

The threshold proportions were defined as operational criteria, not physiological constants. Changing the 5% criterion for early floral biomass to 3% or 8% shifted the pooled estimate by approximately 80 GDD in either direction, while differences among progenies remained within 34–46 GDD. Thus, the selected proportion affects absolute threshold estimates but not the tests of progeny-specific parameters.

Destructive sampling ended at approximately 2119 GDD. Thresholds beyond this point, specifically 95% of the harvest-index asymptote and 90% of the floral-biomass asymptote, represent model extrapolations and are reported as projections.

Relationships between morphometric descriptors and biomass were modeled separately for each S_1_ progeny, using total or floral dry biomass as the response and individual descriptors as predictors, to assess their potential for non-destructive biomass estimation. Given the limited number of progenies, separate models were fitted with progeny treated as a fixed grouping factor. Relationships involving plant height, basal stem diameter (D1), and crown diameters (dc1, dc2) approached saturation and were fitted using logistic functions. Biomass was normalized by its maximum within each progeny (*K*), and the resulting proportion was modeled using a generalized linear model with a logit link. Predicted biomass was calculated using Equation (4):

(4) $$\mathrm{Predicted}\;\mathrm{biomass}\;=\;\frac{K}{1\;+\;\exp\left[-\left(a\;+\;b\;x\right)\right]}$$

where *a* and *b* are the intercept and slope on the logit scale, respectively, and *x* is the descriptor value. Remaining descriptors were analyzed by ordinary least-squares linear regression. Because the logistic response represented a continuous proportion, dispersion was estimated from the data (quasi-binomial) instead of being fixed at 1. Model *p*-values were adjusted using the Holm method [51] within each response (42 tests; Table A4). These models describe empirical size–biomass relationships across sampling dates, whereas the complementary GDD-adjusted analysis evaluates information beyond thermal time. Logistic model fit was expressed as deviance-based pseudo-R^2^ (1 − residual deviance/null deviance), while linear models used conventional R^2^. These measures are not directly comparable and were interpreted qualitatively.

Fixing K reduces the number of estimated parameters but derives the asymptote from the same dataset, making logistic fit statistics conditional on this value. For each descriptor, the null hypothesis of a zero effect was tested using an F test for linear regression slopes or a *t* test for logistic regression coefficients with estimated dispersion.

Harvest index was compared among progenies using two-way ANOVA with progeny, sampling week, and their interaction as fixed effects, restricted to reproductive-phase samplings with floral biomass present in every plant (weeks 8–14; *n* = 35 per progeny). Median-centered Levene’s test across the 21 progeny × week groups showed no significant variance heterogeneity (*p =* 0.20). Given the lower variance in week 8, the progeny effect was also assessed using HC3 robust standard errors and generalized least squares with week-specific variances, both confirming significance (*p* < 0.001). Week-adjusted means were compared using Tukey’s HSD and the residual mean square of the full model. Organ biomass and biomass fractions at week 14 were compared using one-way ANOVA for each organ, with Holm adjustment across the four organs. Tukey’s HSD was applied only where the Holm-adjusted ANOVA was significant. 

Two complementary analyses were conducted. First, progeny differences were assessed through nested log-logistic models sharing a common slope *b*. For the half-maximum parameter, the full model with progeny-specific *d* and *e* was compared with a restricted model sharing *e*; for the asymptote, the comparison used a restricted model sharing *d*. Hypotheses were tested primarily using HC1-robust Wald tests, with conventional F tests based on residual sums of squares reported for comparison. Both tests evaluated the same nested hypotheses, and *p*-values were Holm-adjusted across six biomass compartments, separately for each test and parameter (Table A2). Pairwise differences in *e* among progenies (Table A3, Appendix A) were summarized using 90% percentile confidence intervals from the same stratified bootstrap.

Second, the incremental contribution of four morphometric descriptors to floral biomass beyond accumulated GDD was evaluated. Floral biomass was regressed on GDD using a pooled linear model, as including progeny did not significantly improve fit (Section 2.4; weeks 8–14, *n* = 105). Each descriptor—plant height, D1, branch number, and mean crown diameter (dc1 and dc2)—was then added separately, as expressed in Equation (5):

*Y_i_* = *β*_0_ + *β*_1_*GDD_i_* + *β*_2_*X_i_* + *ε_i_* (5)

where *Y_i_* is floral dry biomass, GDD_i_ is accumulated thermal time, *X_i_* is the descriptor (omitted from the GDD-only model), and *ε_i_* is the residual error. The *p*-values for the four descriptor effects were Holm-adjusted. Predictive performance was assessed by leave-one-week-out cross-validation, predicting each sampling week from models fitted to the remaining six weeks. Performance was summarized using cross-validated R^2^ and root mean squared error (RMSE; g per plant), with R^2^ calculated across the 105 out-of-week predictions as

(6) $$R_{\mathrm{CV}}^{2}=1-\frac{\sum_{i=1}^{105}{\left(y_{i}-{\hat{y}}_{i}\right)}^{2}}{\sum_{i=1}^{105}{\left(y_{i}-\bar{y}\right)}^{2}}$$

where *ŷ_i_* is the prediction from the model excluding plant i’s sampling week, and *ȳ* is the mean of the 105 observed values. Within-week Pearson correlations between each descriptor and total or floral biomass were calculated and averaged across weeks.

All analyses and figures were generated using R 4.5.1 [52], with ggplot2 4.0.3 [53], patchwork 1.3.2, ragg 1.5.2, dplyr 1.2.1, tidyr 1.3.2, readxl 1.5.0, readr 2.2.0, lubridate 1.9.5, and multcompView 0.1-12. Three-parameter log-logistic models were fitted by nonlinear least squares using drc 3.0-1 [54], with Figure 3 curves generated from equivalent nls() fits. Linear and generalized linear models, ANOVA, Tukey’s HSD, Holm adjustment, bootstrap resampling, and HC covariance matrices were implemented using the base stats and parallel packages; generalized least squares used nlme 3.1-168, and Levene’s test and HC3 ANOVA used car 3.1-5.

## 5. Conclusions

In this single-site, single-season study, organ dry biomass in three S_1_ progenies derived from photoperiod-sensitive medicinal *Cannabis sativa* L. cultivars followed sigmoidal trajectories with accumulated growing degree days (GDDs). Progeny-specific half-maximum parameters did not significantly improve model fit, although equivalent developmental timing cannot be inferred. Robust tests identified asymptote differences only for stems, leaves, and roots. DOC exhibited a higher harvest index than PW and SCH despite no significant difference in final floral biomass, highlighting the distinction between reproductive allocation and absolute yield. Morphometric descriptors were associated with biomass across the crop cycle, although they added limited information on floral biomass beyond GDDs, requiring independent validation for predictive applications. The estimated thermal thresholds were conditional on the greenhouse-to-field transition and temperature-calculation method, limiting their generalization. As floral biomass and harvest index continued to increase at the final sampling, and thresholds beyond the sampled period (Table 1) are projections, physiological maturity and optimal harvest timing remained undetermined. Further validation across seasons and environments, supported by improved temperature measurements and assessments of cannabinoid yield and maturity, is needed to inform breeding and crop management.

## Figures and Tables

**Table 1:** Thermal-time thresholds (GDD) for developmental progression in the three S_1_ progenies, estimated using LL.3 models. Values are point estimates with 95% bootstrap confidence intervals (Section 4.5). DOC, PW and SCH, S_1_ progenies derived from the cultivars Doctor, Purple Wreck and Schanti, respectively; Pooled, common model fitted to the three progenies (*n* = 210); CI, confidence interval. † Upper confidence limits constrained by the model-fitting bound (Section 2.2).

Developmental Phase	Threshold Criterion	DOC (95% CI)	PW (95% CI)	SCH (95% CI)	Pooled Estimate (95% CI)
Early biomass accumulation	0.5% of total-biomass asymptote	632 (514–742)	586 (497–678)	550 (438–707)	582 (520–652)
Onset of active vegetative growth	5% of total-biomass asymptote	906 (850–975)	881 (827–941)	840 (777–942)	871 (834–914)
Half-maximal stem biomass	50% of stem-biomass asymptote	1325 (1296–1371)	1337 (1300–1381)	1326 (1266–1490)	1328 (1298–1370)
Early floral biomass	5% of floral-biomass asymptote	1421 (1358–2004) †	1427 (1407–1482)	1455 (1417–1672)	1424 (1402–1521)
Onset of active floral biomass accumulation	10% of floral-biomass asymptote	1561 (1462–2261) †	1522 (1500–1619)	1577 (1523–1858)	1542 (1505–1678)
Half-maximal floral biomass	50% of floral-biomass asymptote	2057 (1751–3233) †	1838 (1739–2100)	1996 (1837–2526)	1946 (1824–2243)
Harvest index at 95% of asymptote (projected)	95% of harvest-index asymptote	2225 (2033–2485)	2221 (2080–2427)	2324 (2125–2594)	2262 (2154–2395)
Floral biomass at 90% of asymptote (projected)	90% of floral-biomass asymptote	2710 (2077–4629) †	2221 (1995–2734)	2527 (2197–3434)	2455 (2203–2997)

**Figure A1:**
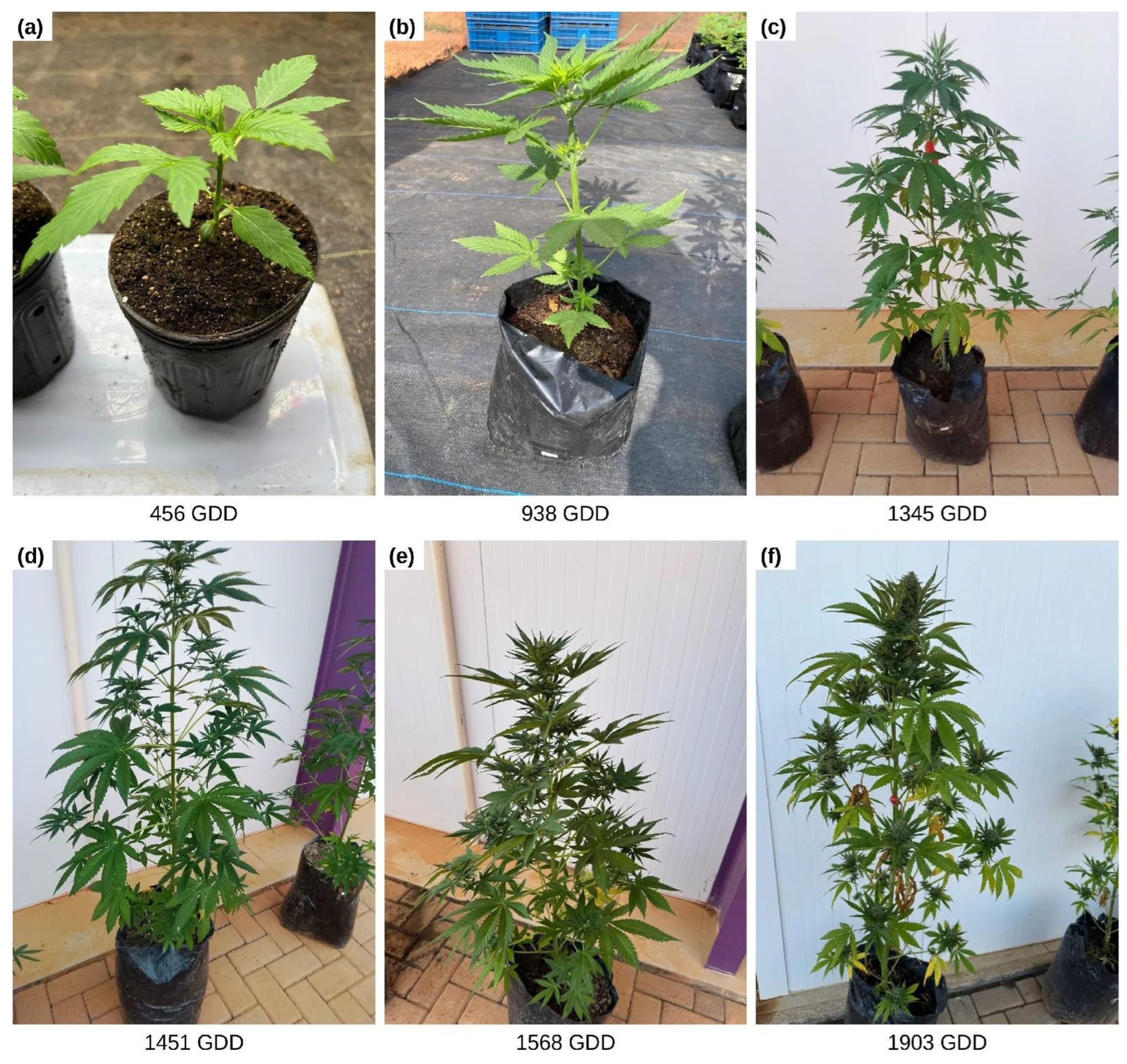
Plants of the S_1_ progenies of *Cannabis sativa* L. photographed at six of the sampling dates shown in Figure 2b: (**a**) 456 GDD, end of propagation (sampling 1; 1 L pot); (**b**) 938 GDD (sampling 4); (**c**) 1345 GDD (sampling 7); (**d**) 1451 GDD (sampling 8); (**e**) 1568 GDD (sampling 9); (**f**) 1903 GDD (sampling 12). Plants in (**b**–**f**) were grown in 19 L bags. No photograph is available for the final sampling (2119 GDD). Panels are not to a common scale.

## Data Availability

The original contributions presented in this study are included in the article and its appendices. Further inquiries can be directed to the corresponding author.

## Abbreviations

The following abbreviations are used in this manuscript:

ANOVA	Analysis of variance
APEPI	Associação de Apoio à Pesquisa e Pacientes de Cannabis Medicinal
BT	Total plant dry biomass
CBD	Cannabidiol
CI	Confidence interval
dc1, dc2	Crown diameters (first and second measurement axes)
D1	Basal stem diameter
D2	Internode stem diameter
DOC	S_1_ progeny of the cultivar Doctor
DTT	Daily thermal time
GDDs	Growing degree days
HI	Harvest index
HSD	Honestly significant difference
INMET	Instituto Nacional de Meteorologia (Brazilian National Institute of Meteorology)
LED	Light-emitting diode
LL.3	Three-parameter log-logistic model
PW	S_1_ progeny of the cultivar Purple Wreck
RMSE	Root mean squared error
S_1_	First selfed generation
SCH	S_1_ progeny of the cultivar Schanti
STS	Silver thiosulfate
*Tb*	Base temperature
*Tc*	Ceiling temperature
THC	Δ9-Tetrahydrocannabinol
UPOV	International Union for the Protection of New Varieties of Plants

## Appendix A

**Table A1:** Phases of the GDD-based phenological scale proposed for the S_1_ progenies and their correspondence with the decimal code for hemp growth stages [29]. In that system, 10xx is the nth leaf pair, 2001 flower primordia, 2200 the first pistillate flowers, 2201 the first visible styles, and 2202 50% of bracts formed; the suffix b marks the same stage without seed, as in all plants of this study [24]. Observed values are the sampling dates at which a stage was first recorded; model thresholds are pooled LL.3 estimates (Table 1); projected values lie beyond the last sampling (~2119 GDD). * Boundary set by management. † Codes 2203–2205 (seed maturity) do not apply to unpollinated plants; senescence (principal stage 3) was not assessed.

Phase	Decimal Code [29]	Description	Start, Observed (GDD)	Start, Model Threshold (GDD)
Propagation (Prop)	0000–0003	Germination, emergence and seedling establishment	0	—
Early vegetative (Veg I)	10xx	Establishment after transfer to cultivation bags	456 *	—
Active vegetative growth (Veg II)	10xx	Pronounced vegetative growth	—	871 (5% of total-biomass asymptote)
Transition (not a phase)	2001–2201	Flower primordia and first solitary pistillate flowers under the 20-h photoperiod	938–1072	—
Flowering phase 1 (FP1)	2201–2202b	Condensed inflorescences after the transfer to short days; onset of floral biomass accumulation	1345 (inflorescences); 1451 (floral biomass)	1424 (5%); 1542 (10% of floral-biomass asymptote)
Flowering phase 2 (FP2)	2202b	Pronounced floral biomass accumulation, an operational subdivision within stage 2202b (50% of bracts formed), which was recorded from 1451 GDD; 95% of the harvest-index asymptote projected at 2262 GDD	—	1946 (50% of floral-biomass asymptote)
Flowering phase 3 (FP3)	— †	Late floral biomass accumulation	—	2455 (90% of floral-biomass asymptote, projected)

**Table A2:** Tests of progeny-specific half-maximum (*e*) and asymptote (*d*) parameters in nested three-parameter log-logistic models with a common slope *b* for six biomass compartments of three S_1_ progenies of *Cannabis sativa* L. (n = 210). For *e*, a full model with progeny-specific *d* and *e* is compared with a model with a common *e*; for *d*, the same full model is compared with a model with a common *d*. Wald tests use heteroscedasticity-consistent (HC1) standard errors (χ^2^, 2 df) and are the primary inference; F tests (2 and 203 df) assume constant residual variance. *p*-values are Holm-adjusted across the six compartments, separately for each test and parameter.

Compartment	Half-Maximum Parameter *e*				Asymptote *d*			
	Wald χ^2^ (HC1)	*p* (Holm)	F	*p* (Holm)	Wald χ^2^ (HC1)	*p* (Holm)	F	*p* (Holm)
Total biomass	0.89	1.000	0.58	1.000	6.57	0.112	4.80	0.028
Above-ground biomass	0.77	1.000	0.55	1.000	5.77	0.112	4.03	0.038
Stem	0.12	1.000	0.09	1.000	42.41	<0.001	22.95	<0.001
Leaf	2.71	1.000	1.34	1.000	27.47	<0.001	15.30	<0.001
Floral biomass	1.26	1.000	1.11	1.000	3.12	0.210	3.24	0.041
Root	0.46	1.000	0.31	1.000	15.31	0.002	12.12	<0.001

**Table A3:** Parameter estimates of three-parameter log-logistic (LL.3) models of organ-specific biomass accumulation and harvest index as a function of accumulated growing degree days (GDDs) in three S_1_ progenies of *Cannabis sativa* L. *b*, slope parameter; *d*, fitted upper asymptote (g, or dimensionless for harvest index); *e*, half-maximum parameter (GDD at 50% of d). Heteroscedasticity-consistent (HC1) standard errors in parentheses, from the fit to each progeny; they are not the basis of the bootstrap intervals in Table 1. Organ-specific curves were fitted independently, so their asymptotes should not be summed to estimate whole-plant biomass. R^2^ is calculated as 1 − SSres/SStot; RSE is the residual standard error, √(SSres/(*n* − 3)), on the scale of the response.

Organ	Progeny	*b* (SE)	*d* (SE)	*e* (SE), GDD	*n*	R^2^	RSE
Total biomass	DOC	−6.54 (1.34)	58.28 (7.48)	1421 (74)	70	0.918	6.30
Total biomass	PW	−5.76 (0.79)	71.79 (6.79)	1468 (61)	70	0.935	6.33
Total biomass	SCH	−5.54 (1.15)	76.21 (9.23)	1428 (95)	70	0.889	9.12
Root biomass	DOC	−17.15 (10.32)	6.38 (0.23)	1254 (34)	70	0.878	1.02
Root biomass	PW	−9.68 (2.54)	5.56 (0.38)	1230 (28)	70	0.829	1.03
Root biomass	SCH	−10.86 (3.08)	7.08 (0.34)	1206 (32)	70	0.797	1.48
Stem biomass	DOC	−11.15 (2.42)	12.42 (0.66)	1325 (21)	70	0.911	1.64
Stem biomass	PW	−8.48 (0.98)	16.77 (0.75)	1337 (24)	70	0.920	1.96
Stem biomass	SCH	−8.22 (2.69)	19.56 (1.71)	1326 (55)	70	0.833	3.49
Leaf biomass	DOC	−11.55 (1.66)	15.32 (0.61)	1171 (26)	70	0.837	2.77
Leaf biomass	PW	−8.95 (1.60)	20.42 (0.88)	1190 (26)	70	0.874	3.06
Leaf biomass	SCH	−10.41 (1.65)	19.29 (0.88)	1130 (35)	70	0.757	4.37
Floral biomass	DOC	−7.97 (2.46)	45.97 (38.56)	2057 (409)	70	0.903	2.84
Floral biomass	PW	−11.62 (2.06)	28.81 (5.85)	1838 (79)	70	0.949	2.01
Floral biomass	SCH	−9.32 (1.45)	46.01 (13.00)	1996 (137)	70	0.954	2.15
Harvest index	DOC	−11.43 (1.53)	0.520 (0.047)	1720 (44)	70	0.965	0.035
Harvest index	PW	−12.32 (1.25)	0.448 (0.038)	1749 (33)	70	0.979	0.023
Harvest index	SCH	−11.09 (1.38)	0.507 (0.050)	1782 (50)	70	0.970	0.029

**Table A4:** Parameter estimates of descriptor-based models for total biomass (roots included) and for floral biomass in three S_1_ progenies of *Cannabis sativa* L. (DOC, PW, SCH). *n* = 70 per descriptor and progeny in every model.

Descriptor	Model	Total Biomass (Roots Included)				Floral Biomass			
		Intercept	Coefficient	*p* (Holm)	R^2^	Intercept	Coefficient	*p* (Holm)	R^2^
Plant height—DOC	logistic	−4.919	0.066	<0.001	0.819	−7.743	0.089	<0.001	0.561
Plant height—PW	logistic	−5.651	0.072	<0.001	0.859	−11.046	0.121	<0.001	0.643
Plant height—SCH	logistic	−4.921	0.062	<0.001	0.858	−7.429	0.076	<0.001	0.502
Basal stem diameter (D1)—DOC	logistic	−5.085	0.449	<0.001	0.852	−7.952	0.600	<0.001	0.590
Basal stem diameter (D1)—PW	logistic	−5.356	0.515	<0.001	0.849	−8.577	0.691	<0.001	0.629
Basal stem diameter (D1)—SCH	logistic	−4.589	0.379	<0.001	0.825	−6.523	0.422	<0.001	0.531
Internode stem diameter (D2)—DOC	linear	−11.083	9.389	0.008	0.168	−11.151	4.362	0.002	0.209
Internode stem diameter (D2)—PW	linear	−14.936	9.886	<0.001	0.251	−7.932	3.069	0.004	0.187
Internode stem diameter (D2)—SCH	linear	9.586	5.861	0.626	0.054	1.157	1.389	1.000	0.022
Internode length—DOC	linear	23.933	0.083	1.000	0.004	5.843	0.017	1.000	0.001
Internode length—PW	linear	27.205	0.079	1.000	0.004	5.176	0.024	1.000	0.003
Internode length—SCH	linear	38.074	−0.088	1.000	0.004	7.744	−0.017	1.000	0.001
Leaflet number (leaf 1)—DOC	linear	−7.163	4.872	0.013	0.154	−0.370	0.975	1.000	0.036
Leaflet number (leaf 1)—PW	linear	−34.960	8.793	<0.001	0.415	−7.099	1.788	0.039	0.133
Leaflet number (leaf 1)—SCH	linear	−17.348	6.396	0.028	0.135	4.884	0.261	1.000	0.002
Leaflet length (leaf 1)—DOC	linear	37.739	−0.124	1.000	0.019	8.365	−0.022	1.000	0.004
Leaflet length (leaf 1)—PW	linear	38.222	−0.075	1.000	0.008	8.494	−0.022	1.000	0.005
Leaflet length (leaf 1)—SCH	linear	14.505	0.205	1.000	0.031	1.947	0.052	1.000	0.015
Leaflet width (leaf 1)—DOC	linear	66.832	−1.883	<0.001	0.403	16.189	−0.459	0.032	0.138
Leaflet width (leaf 1)—PW	linear	78.141	−1.799	<0.001	0.374	18.590	−0.468	0.003	0.196
Leaflet width (leaf 1)—SCH	linear	86.512	−2.594	<0.001	0.423	18.541	−0.573	0.020	0.152
Petiole length (leaf 1)—DOC	linear	2.309	0.680	<0.001	0.227	−3.780	0.284	<0.001	0.229
Petiole length (leaf 1)—PW	linear	15.078	0.322	0.089	0.103	0.207	0.124	0.070	0.118
Petiole length (leaf 1)—SCH	linear	22.239	0.275	0.856	0.045	3.807	0.073	1.000	0.023
Leaflet number (leaf 2)—DOC	linear	−11.029	5.427	0.006	0.176	−1.058	1.074	1.000	0.040
Leaflet number (leaf 2)—PW	linear	−41.683	9.564	<0.001	0.486	−10.081	2.158	0.004	0.191
Leaflet number (leaf 2)—SCH	linear	−10.708	5.602	0.071	0.110	6.583	0.050	1.000	0.000
Leaflet length (leaf 2)—DOC	linear	37.355	−0.119	1.000	0.020	9.656	−0.036	1.000	0.011
Leaflet length (leaf 2)—PW	linear	39.812	−0.091	1.000	0.012	9.352	−0.031	1.000	0.010
Leaflet length (leaf 2)—SCH	linear	11.217	0.240	0.856	0.043	0.102	0.072	1.000	0.028
Leaflet width (leaf 2)—DOC	linear	61.690	−1.606	<0.001	0.314	13.832	−0.341	0.279	0.082
Leaflet width (leaf 2)—PW	linear	76.577	−1.734	<0.001	0.368	18.218	−0.453	0.004	0.194
Leaflet width (leaf 2)—SCH	linear	90.623	−2.833	<0.001	0.431	19.055	−0.606	0.025	0.145
Petiole length (leaf 2)—DOC	linear	3.012	0.653	0.001	0.210	−3.214	0.265	0.003	0.200
Petiole length (leaf 2)—PW	linear	14.301	0.347	0.068	0.113	0.516	0.121	0.109	0.106
Petiole length (leaf 2)—SCH	linear	24.747	0.228	1.000	0.026	3.456	0.085	1.000	0.027
Crown diameter (dc1)—DOC	logistic	−3.250	0.074	<0.001	0.842	−5.729	0.105	<0.001	0.569
Crown diameter (dc1)—PW	logistic	−2.592	0.069	<0.001	0.792	−5.815	0.114	<0.001	0.765
Crown diameter (dc1)—SCH	logistic	−2.725	0.080	<0.001	0.783	−4.641	0.096	<0.001	0.450
Crown diameter (dc2)—DOC	logistic	−3.204	0.078	<0.001	0.874	−6.059	0.118	<0.001	0.646
Crown diameter (dc2)—PW	logistic	−2.608	0.077	<0.001	0.805	−5.292	0.113	<0.001	0.702
Crown diameter (dc2)—SCH	logistic	−2.593	0.080	<0.001	0.752	−4.435	0.095	<0.001	0.451

Model type indicates the functional form fitted for each descriptor: logistic models (quasi-binomial with logit link fitted to biomass divided by its maximum within each progeny, K) for height, stem diameter (D1) and crown diameters (dc1, dc2); and simple linear models for the remaining descriptors. Intercepts and coefficients are expressed on the scale of each model and are therefore not directly comparable across model types. *p*-values test the null hypothesis that the descriptor term is zero: the regression slope, by F test, in the linear models, and the rate parameter, by *t* test with the dispersion parameter estimated from the data (quasi-binomial), in the logistic models; all *p*-values are Holm-adjusted within each response (42 tests). After adjustment, 27 of the 42 descriptor terms were significant for total biomass and 23 of the 42 for floral biomass. For the linear models, which contain a single predictor, adjusted R^2^ differs from R^2^ by less than 0.02 and is not reported. For the logistic models, R^2^ is a deviance-based pseudo-R^2^ (1 − residual deviance/null deviance); for the linear models it is the ordinary R^2^.

**Table A5:** Sampling dates, accumulated growing degree days (GDDs) and growth stage in the S_1_ progenies. GDD accumulation started on 10 July 2024; values are cumulative up to the day before each sampling. The decimal code [29] gives the range recorded among the sampled plants; suffix b denotes the same stage in plants that do not form seed [24].

Sampling	Date (2024)	Days from 10 July	GDD (°C d)	Decimal Code [29]
1	1 August	22	456.3	1004–1006
2	8 August	29	597.0	1006–1008
3	15 August	36	762.0	1008–1010
4	22 August	43	937.5	1008–1012; 2200
5	29 August	50	1071.8	2200–2201
6	5 September	57	1237.1	2201
7	12 September	64	1345.1	2201–2202b
8	19 September	71	1451.4	2202b
9	26 September	78	1567.5	2202b
10	3 October	85	1687.9	2202b
11	10 October	92	1795.7	2202b
12	17 October	99	1903.1	2202b
13	24 October	106	2008.3	2202b
14	31 October	113	2119.5	2202b
